# Intranasal Oxytocin and Physical Intimacy for Dermatological Wound Healing and Neuroendocrine Stress: A Randomized Clinical Trial

**DOI:** 10.1001/jamapsychiatry.2025.3705

**Published:** 2025-11-12

**Authors:** Ekaterina Schneider, Cristóbal Hernández, Robert Brock, Monika Eckstein, Guy Bodenmann, Markus Heinrichs, Ulrike Ehlert, Severin Läuchli, Beate Ditzen

**Affiliations:** 1 Institute of Medical Psychology, University Hospital, Heidelberg, Germany; 2 Ruprecht-Karls University Heidelberg, Heidelberg, Germany; 3 DZPG (German Center for Mental Health), Partner Site Mannheim, Heidelberg, Ulm, Germany; 4 Escuela de Psicología, Universidad Adolfo Ibáñez, Santiago, Chile; 5 Universidad de los Andes, Chile, Facultad de Ciencias Sociales, Escuela de Psicología, Santiago, Chile; 6 Department of Pediatrics, University Hospital Cologne and Faculty of Medicine, University of Cologne, Cologne, Germany; 7 Department of Psychology, University of Zurich, Zurich, Switzerland; 8 Department of Psychology, University of Freiburg, Freiburg, Germany; 9 Dermatological Clinic, University Hospital Zurich, Zurich, Switzerland

## Abstract

**Importance:**

Close social relationships are linked to improved individual health and even longevity. These effects are hypothesized to be mediated through improved neuroendocrine and immune functioning, particularly in individuals who engage in positive and affectionate interactions. However, systematic data examining these factors in humans are currently lacking.

**Objective:**

To investigate the interacting effects of repeated intranasal oxytocin administration, a behavioral microintervention, and daily physical intimacy on neuroendocrine stress responses and dermatological wound healing.

**Design, Setting, and Participants:**

This randomized clinical trial was a double-blind, randomized, placebo-controlled study whereby participants completed 3 laboratory visits and a 5-day ecological momentary assessment (EMA). During the first laboratory visit, participants received 4 small suction-blister wounds applied to their forearms. Data were collected from November 20, 2011, to July 25, 2013; final analyses were conducted from December 2023 to February 2025.

**Interventions:**

Over the following 7 days, participants were instructed to self-administer either oxytocin or a placebo twice daily and to engage in structured positive interaction (Partner Appreciation Task [PAT]) up to 3 times in total or not.

**Main Outcomes and Measures:**

Wound healing was assessed at 24 hours and 7 days after wounding. Throughout the week, participants collected saliva samples for cortisol analyses and reported their stress levels and experiences of partner interaction 6 times per day (5760 measurement points in total).

**Results:**

The volunteer sample was 80 healthy, heterosexual couples (N = 160 participants, mean [SD] age, 27.6 [5.0] years). Couples in the PAT condition who received daily oxytocin showed improved wound healing (*b* = −0.125, *t*_286_ = −1.983; *P* = .048). However, these effects were not consistently robust in sensitivity analyses (*b* = −0.090, *t*_282_ = −1.643; *P* = .10). Notably, the administration of oxytocin combined with daily affectionate touch (*b* = −0.038, *t*_137_ = −2.091; *P* = .04) and sexual activity (*b* = −0.145, *t*_137_ = −2.122; *P* = .04) was linked to a reduction in wound severity. These associations remained largely consistent in sensitivity analyses (affectionate touch: *b* = –0.037, *t*_135_ = –2.057; *P* = .04; sexual activity: *b* = –0.131, *t*_135_ = –1.900; *P* = .06). Additionally, greater sexual activity was associated with reduced daily cortisol levels (*b* = −0.083, *t*_488_ = −2.813; *P* = .005).

**Conclusions and Relevance:**

This study found that intimate physical contact can reduce cortisol responses and, along with oxytocin administration, promote wound healing. These findings provide a foundation for future interventions that integrate relationship dynamics and neurohormonal modulation to improve health and recovery from illness.

**Trial Registration:**

ClinicalTrials.gov Identifier: NCT01594775

## Key Points

**Question:**

Does the repeated administration of oxytocin, combined with positive couple interaction, improve wound healing?

**Findings:**

In a randomized clinical trial involving 80 romantic couples, those who received daily oxytocin and were instructed to engage in positive interactions showed enhanced healing of dermatological wounds, especially when engaging in daily physical intimacy.

**Meaning:**

These findings suggest that combining oxytocin administration with positive relational behaviors may support physical healing, offering a potential pathway for psychosocial interventions in health-related contexts.

## Teaser

This randomized clinical trial examines the effects of repeated intranasal oxytocin administration and a behavioral microintervention on neuroendocrine stress responses and dermatological wound healing.

## Introduction

Being socially integrated and in a relationship has been linked to improved physical health and reduced mortality rates.^1^ Long-lasting romantic relationships, in particular, can provide security, meaning, and emotional enrichment, and “love” has even been proposed as a factor for improving psychobiological outcomes and public mental health.^2^ Several studies suggest that the quality of a romantic relationship is an even better predictor of health outcomes than simply being in a relationship.^3,4^ Together, this research suggests that it is the repeated supportive, affectionate, and intimate interactions in close relationships that can improve immune functioning and thereby health and longevity.^5,6^

Physical contact is a key form of social support, especially during stress.^7^ Experimental studies (included in reviews^6,8,9^) and ecological momentary assessments (EMAs) in daily life consistently show that affectionate touch enhances well-being by reducing autonomic, neuroendocrine, cortisol, and subjective stress responses.^10,11,12^

Building on these associations, interventions that foster relationship quality likewise benefit both relational and health outcomes,^13^ with cognitive-behavioral approaches showing strong efficacy.^14^ However, most clinical research has targeted specific populations (eg, cancer,^15^ chronic illnesses,^16^ or impaired mental health^17^), leaving preventive couple-based interventions for the general population comparatively understudied. Here, core elements, such as positive attributions and affirmative partner feedback, are empirically supported intervention strategies^18^ and may also affect health.

Wound healing serves as a clinically relevant marker of physical health and immune function, as the body constantly experiences minor physiological injuries requiring repair. Efficient healing in healthy individuals thus reflects adaptive capacity to physiological challenges. Consistent with this, stress has been shown to delay wound healing.^19,20^ Studying wound healing as a physical health outcome and proxy for a healthy immune system can provide insight into the impact of social interactions on health. More specifically, research conducted by Kiecolt-Glaser et al^21^ has demonstrated that marital conflicts can lead to impaired wound healing, altered immune responses, and stress hormone release (for a review, see Kiecolt-Glaser^22^).

The neuropeptide oxytocin is considered a mediator for the positive effects of affectionate interactions on physical and psychological well-being. Some more recent studies link affectionate touch with increases in endogenous oxytocin in animals^23^ and in humans (blood^24,25^ and saliva^12^). Exogenous oxytocin increased touch sensitivity^26^ and reduced stress responses^27^ (for a review, see Cardoso et al^28^). Animal studies suggest that oxytocin improves wound healing,^29,30^ although findings are mixed.^31^ Our recent analysis from the present dataset suggests that intranasal oxytocin affected immune factors in skin wounds immediately and the day after wounding,^32^ but in humans only 1 study has linked endogenous oxytocin levels to both positive partner communication and better wound healing as the main outcome.^33^ To date, no study has tested whether oxytocin administration improves wound healing in humans.

To address this gap, we conducted a double-blind, randomized, placebo-controlled study investigating repeated intranasal oxytocin administration combined with structured positive interaction (Partner Appreciation Task [PAT]) in romantic couples. We hypothesized that oxytocin and positive couple interaction, including both instructed behavior and naturally occurring intimacy, would attenuate psychobiological stress responses and accelerate wound healing. Exploratorily, we also examined associations of affectionate touch and sexual activity with stress regulation.

## Methods

### Participants

A total of 80 healthy heterosexual couples (N = 160) participated in a preregistered clinical trial on oxytocin, couple interaction, and wound healing at University Hospital Zurich, Switzerland (ClinicalTrials.gov identifier NCT01594775). Recruitment was conducted via flyers, online advertisements, brochures, social media, and university mailing lists. Eligibility criteria included age 21 to 45 years and a stable relationship of at least 1 year. Exclusion criteria comprised pregnancy, breastfeeding, acute or chronic physical or mental disorders, extensive skin disease, artificial UV exposure within 3 months, use of medication (except hormonal contraceptives), drug use or alcohol misuse (intake ≥60 g/7.5 dL wine or 2 L beer), or smoking more than 5 cigarettes per day.

The study was approved by the Canton of Zurich ethics committee and monitored by the Clinical Trials Center Zurich. Written informed consent was obtained from all participants, who received 500 CHF (US$629) compensation.

Initial institutional review board approval was granted in 2010 (all amendments approved in October 2011). The study was registered on the University Hospital’s Clinical Trials Register in October 2011 and on ClinicalTrials.gov in May 2012 (see trial protocol in Supplement 1). Data were collected from November 20, 2011, to July 25, 2013.

Hormone data cleaning and analysis occurred from 2012 to 2014. Wound scaling and scoring were completed after laboratory relocation (2016-2018). Exploratory confocal microscopy (2018-2020) was excluded because of insufficient quality; therefore, only standardized photographs of upper skin layers were analyzed. Final statistical analyses were conducted from December 2023 to February 2025.

### Randomization of Drug Administration and Couple Interaction

**Figure 1.**
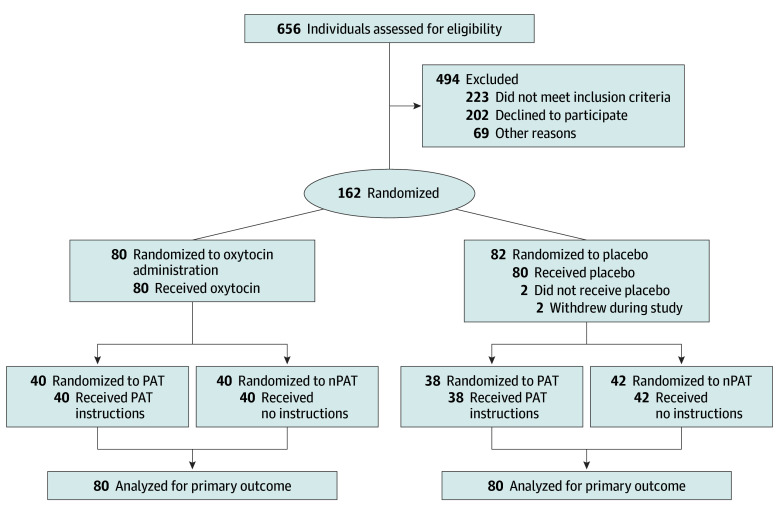
Flow Diagram PAT indicates structured positive interaction (Partner Appreciation Task); nPAT, no instructions.

The couples were randomly assigned, in a double-blind design, to 1 of 4 groups: oxytocin vs placebo treatment and instructed PAT vs no instructions (control [nPAT]) (Figure 1). Randomization was conducted by an independent assistant using the Research Randomizer program. Within each group, half of the women were naturally cycling and half used hormonal contraception, based on information obtained during a prestudy phone screening.^32^

### Procedure

Couples completed 3 laboratory visits across 1 week. At visit 1, participants completed questionnaires, provided urine samples (to exclude drug use and pregnancy), and received standardized blister wounds on the forearms, after which they self-administered oxytocin or placebo nasal spray. Half of the couples received PAT instructions while the others did not. On day 2, participants began the 5-day EMA and at visit 2 (24 hours after wounding) underwent a second wound assessment. At visit 3 (day 7), wound healing was reassessed.

### Blister Wound Application and Wound Healing Ratings

Blister wounds were applied to the participants’ forearms according to established protocols.^21,34^ Blisters were induced on the volar forearms by shaving and cleaning the skin, applying a suction blister template, and using vacuum (−80 mm Hg; Scientific GmbH) plus heat to separate the dermal-epidermal junction, yielding four 0.7-cm blisters. Wound fluid was drained, and the epidermis removed from 2 blisters at visit 1 and the remaining 2 at visit 2 (24 hours later). All wounds were covered with hydrocolloid bandages to be kept for 3 days before plaster change. Confocal laser microscopy images were obtained at 1 hour (T1), 24 hours (T2), and 7 days (T3) after wounding at standardized distances (eFigure in Supplement 2). Wound severity was rated by 2 independent raters (intraclass correlation = 0.821; 95% CI, 0.754-0.870; *F*_159,159_ = 5.772; *P* < .001) using a revised Photographic Wound Assessment Tool (revPWAT).^35^ For the blister wounds, not all criteria of revPWAT were applicable and only 4 factors were applied: (1) granulation tissue type, (2) granulation tissue amount, (3) wound edges, and (4) skin variability (erythema, encrustation, pigmentation). Each factor was rated 0 to 4 and summed for a maximum severity score of 16. Although the institutional review board protocol and trial registration defined wound healing as an overall reduction in wound size, we opted for this revPWAT-based scoring approach, as it allows for a more detailed and clinically meaningful assessment of wound severity than wound size alone.

### Intranasal Oxytocin Administration and PAT

Approximately 50 minutes after wound application, both partners self-administered a nasal spray containing either oxytocin (Syntocinon; Novartis; 3 puffs/nostril, total dose, 24 IU) or placebo (identical composition without oxytocin). About 95 minutes after wounding (45 minutes after spray), couples were randomized to receive either PAT or a control condition (nPAT). The PAT was a 10-minute structured, video-recorded discussion targeting (1) attention to valued relationship aspects, (2) gratitude and positive expectations, and (3) reciprocal verbal appreciation. A list of 23 positive relationship characteristics (eg, trust, joint planning, dyadic coping) was provided for rating and discussion, how these applied to the relationship, and what the couple particularly liked about them.^36^ Previous work indicates the PAT has stress-buffering effects in the placebo group of this dataset,^36^ and in other study samples, it increased reward-related brain activity^37^ and improved momentary relationship satisfaction in women with chronic depression.^38^

In the control condition, couples engaged in 10 minutes of free, unstructured interaction while being videotaped. PAT couples were instructed to repeat the task up to 2 additional times during the following week and to document positive aspects via the iDialogPad application, while control couples documented joint time and interactions. All participants received sprays for home use and self-administered oxytocin or placebo (2 puffs/nostril, 32 IU/d) at predetermined times twice daily for 5 days.

### Psychometric Measures

#### Partnership Questionnaire

Relationship quality was assessed with the 30-item German Partnership Questionnaire (PFB), comprising three 10-item subscales: quarreling, tenderness, and communication (responses, 0 = never to 3 = very often).^39^ Internal consistency in our sample was α = .84.

#### NEO-FFI-30

Personality was measured with the German NEO Five Factor Inventory (FFI) 30, a 30-item short form assessing neuroticism, extraversion, openness, agreeableness, and conscientiousness (6 items each; responses from 0 = strong rejection to 4 = strong approval).^40^ Internal consistencies ranged from α = .64 to .81.

### EMA and Cortisol Analyses

For 5 consecutive days, participants completed EMA via iDialogPad (6 prompts/d). At each prompt, participants were asked whether they had interacted with their partner since the last entry. If so, they could select 1 or more types of interactions (eg, conversation, affectionate touch, sexual activity, conflict, or other). Additionally, participants rated their current emotional state, including how stressed vs relaxed they felt on a 5-point Likert scale (0 = very stressed, 4 = very relaxed), and control variables (eating, drinking, sports).

Saliva samples (SaliCaps; IBL) were collected at waking, 30 minutes, 2.5 hours, 8 hours, 12 hours, and bedtime; stored at −20 °C; and assayed for cortisol using a competitive luminescence immunoassay (IBL; coefficient of variation <10%) at the Institute of Psychology’s biomarker laboratory at University of Zurich. Participants self-administered oxytocin/placebo sprays twice daily (8 hours and 12 hours after waking) and performed the positive interaction task twice, documenting both in iDialogPad.

### Statistical Analysis

A priori power analysis for the main outcome wound healing (G*Power,^41^ *F* = 0.40, power = 0.82, α = .05) indicated a minimum of 76 couples; 100 were targeted to account for attrition. Because of minimal dropout (1 couple), the final sample comprised 80 couples. Analyses were conducted in R^42^ using 3-level hierarchical linear models (observations nested within individuals nested within couples). Models controlled for sex and age; cortisol area under the curve with respect to ground (AUCg) analyses additionally controlled for day, body mass index, food and beverage intake, and physical activity. Restricted maximum likelihood estimation was applied; missing data were handled by listwise deletion.^43^ Linear models were estimated with nlme (lme function)^44^ and logistic models with lme4.^45^

#### Effects of Oxytocin Administration and PAT on Wound Healing

To test the effects of oxytocin and PAT on wound healing, revPWAT scores were predicted from time (T1, T2, T3), oxytocin, PAT, and their 3-way interaction. Wound healing values were log-transformed after adding a constant of 1 to normalize residuals, and time-specific variances were modeled. Random intercepts were included for each dyad *u*_0k_ and members within dyads *v*_0jk_.

log(PWAT_tjk_ + 1) = β_0_ + β_1_ Time_t_ + β_2_ Oxytocin_k_ + β_3_ PAT_k_ + β_4_ Sex_jk_ + β_5_ Age_jk_ + β_6_ (Time_t_ × Oxytocin_k_) + β_7_ (Time_t_ × PAT_k_) + β_8_ (Oxytocin_k_ × PAT_k_) + β_9_ (Time_t_ × Oxytocin_k_ × PAT_k_) + *u*_0k_ [Dyad] + *v*_0jk_ [Member within dyad] + ε_tjk_

with

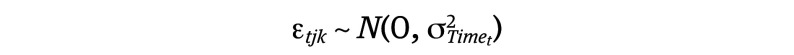
Sensitivity analyses excluded cases with extreme residuals.

#### Effects of Oxytocin Administration and Physical Intimacy in Daily Life on Wound Healing

We analyzed the effects of oxytocin administration and physical intimacy on wound healing: specifically, the number of daily affectionate touch sequences or sexual activities. Totals were calculated by summing dummy-coded EMA reports (yes/no per prompt) per participant. As EMA began on day 2, models predicted wound healing across T2 to T3, controlling for baseline wound severity (T1). Two separate models were estimated (affectionate touch, sexual activity), each controlling for PAT condition, oxytocin administration, sex, and age. Three-way interaction (time × oxytocin × intimacy variable) tested effects on wound healing. Random intercepts were included for each dyad *u*_0k_ and members within dyads *v*_0jk_.

log(PWAT_tjk_ + 1) = β_0_ + β_1_ Time 2-3_t_ + β_2_ BaselinePWAT_jk_ + β_3_ Oxytocin_k_ + β_4_ PAT_k_ + β_5_ Affectionate Touch_jk_
*+ β*_6_ Sex_jk_ + β_7_ Age_jk_ + β_8_ (Time 2-3_t_ × Oxytocin_k_) + β_9_ (Time 2-3_t_ × Affectionate Touch_jk_) + β_10_(Oxytocin_k_ × Affectionate Touch_jk_) + β_11_ (Time_t_ × Oxytocin_k_ × Affectionate Touch_jk_) + *u*_0k_ [Dyad] + *v*_0jk_ [Member within dyad] + ε_tjk_

with

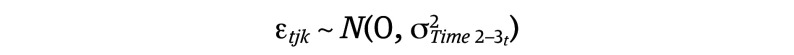
Sensitivity analyses excluded cases with normalized residuals above or below 3 points.

#### Effects of Oxytocin Administration and Physical Intimacy on Psychobiological Stress Responses

To test the second hypothesis, we examined effects of oxytocin and physical intimacy on cortisol and subjective stress. Daily cortisol was quantified as AUCg,^46^ log-transformed to normalize residuals and correct heteroskedasticity. Subjective stress was averaged per day. Separate models predicted AUCg and daily stress from oxytocin administration and the number of affectionate touches or sexual activities.

Exploratory analyses assessed lagged associations between stress, affectionate touch, and sexual activity. Daily stress ratings (lower rating = higher stress) were aggregated, and touch/sexual activity were lagged within day (last value = not applicable). Touch/ sexual activity were predicted by lagged stress, time, sex, and age, with stress person-mean centered. Because of minimal partner-level variance, models nested observations within individuals. Both specifications (with and without partner nesting) converged in the same substantive results.

## Results

### Sample Characteristics

The study included 160 individuals (N = 80 couples; mean [SD] age 27.6 [5.0] years). Mean (SD) relationship duration was 3.96 (2.59) years, with cohabitation lasting a mean (SD) 2.06 (1.80) years. Most participants (92; 57.5%) held a university degree and were employed (127; 79.4%) (eTable 1 in Supplement 2).

### Baseline Comparability

Independent *t* tests revealed no significant differences between oxytocin and placebo groups on relationship variables (relationship quality, PFB score, duration, cohabitation) or personality traits (NEO-FFI-30 score), nor in EMA-reported affectionate touch or sexual activity, indicating balanced groups (eTables 2 and 3 in Supplement 2).

### Adherence to Nasal Spray and PAT

Daily adherence to nasal spray use was high (placebo, 72 [89.9%]; oxytocin, 73 [91.1%]), with low adverse effects (3%-4%). Among PAT participants, 75 (96.2%) completed the task at least once and 53 (68%) on 2 or more days, with similar adherence between groups. Oxytocin did not increase the frequency of instructed positive interactions. When asked, whether they had experienced any effects of the spray, among all 80 couples, only 5 reported any subjective effects, with only 1 of these suspected to have received oxytocin.

### Effects of Oxytocin Administration and PAT on Wound Healing

**Figure 2.**
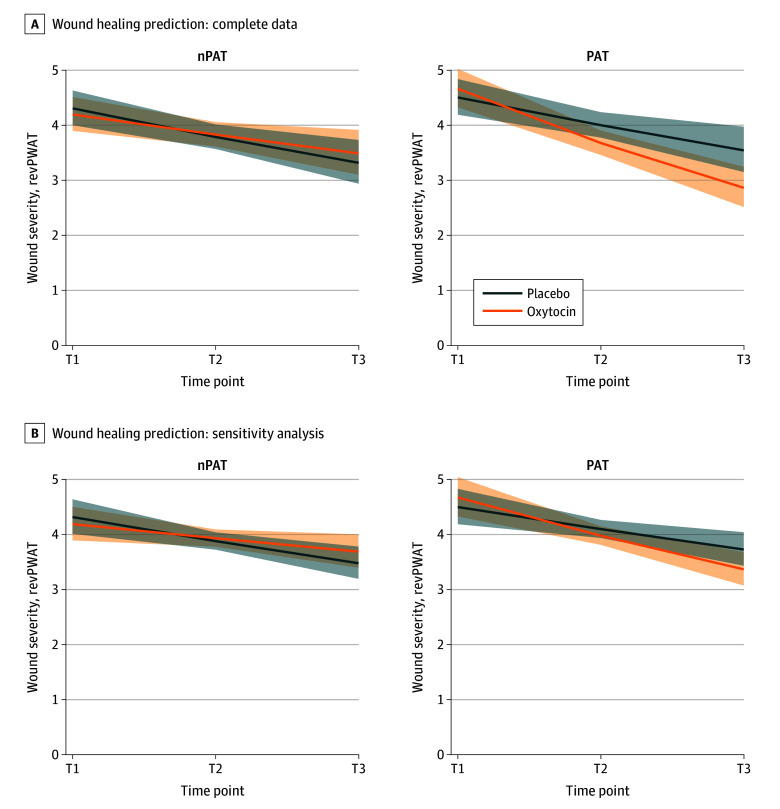
Oxytocin Administration and Partner Appreciation Task (PAT) Predicting Wound Healing A, Interaction effect between oxytocin and PAT in predicting wound healing scores across time points 1 to 3. B, Same model after excluding 2 influential cases. Time T1 is 1 hour after wounding; T2, 24 hours after wounding; and T3, 7 days after wounding. nPAT indicates the group who received no instructions; revPWAT, revised Photographic Wound Assessment Tool.

In the first model, wound healing improved over time (*b* = −0.103, *t*_286_ = −3.278; *P* = .001). Main effects of oxytocin (*b* = −0.021, *t*_76_ = −0.493; *P* = .62) and PAT (*b* = 0.037, *t*_76_ = 0.860, *P* = .39) were nonsignificant, as were the 2-way interactions (time × PAT: *b* = 0.007, *P* = .87; time × oxytocin: *b* = 0.030, *P* = .50; oxytocin × PAT: *b* = 0.049; *P* = .42). A significant 3-way interaction emerged (time × oxytocin × PAT: *b* = −0.125, *t*_286_ = −1.983; *P* = .048), indicating that oxytocin enhanced wound healing under PAT but not nPAT conditions (Figure 2A). Sensitivity analyses identified 2 influential cases; exclusion rendered the effect nonsignificant (b = −0.090, *t*_282_ = −1.643; *P* = .10) though effect direction was consistent (Figure 2B). Additionally, women exhibited lower wound healing than men (*b* = −0.059, *t*_65_ = −4.070; *P* < .001).

### Effects of Oxytocin Administration and Physical Intimacy in Daily Life on Wound Healing

**Figure 3.**
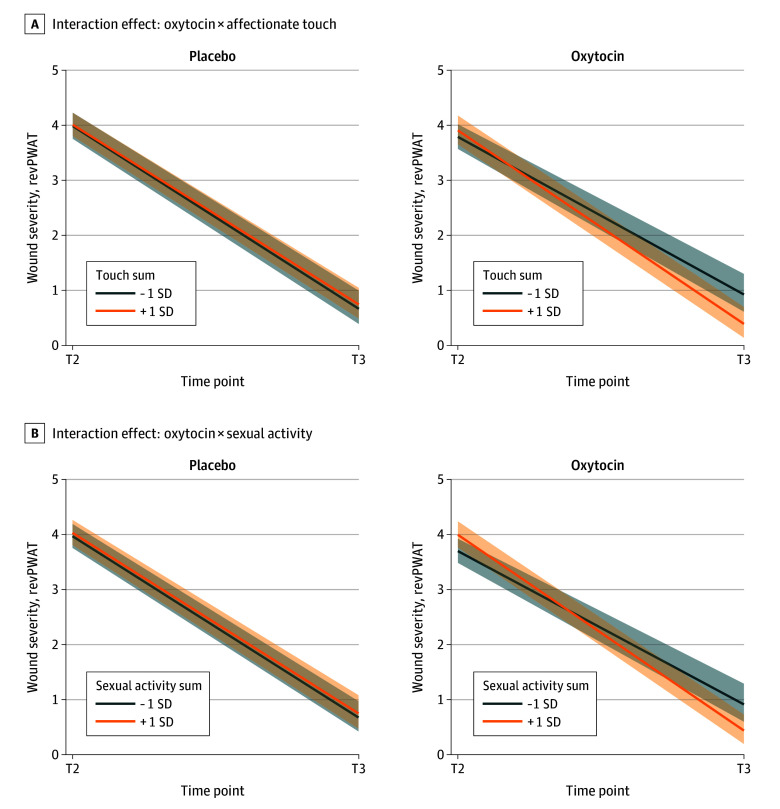
Oxytocin Administration and Physical Intimacy Predicting Wound Healing A, Interaction effect between oxytocin and affectionate touch. B, Interaction effect between oxytocin and sexual activity (−1 SD in red and +1 SD in blue). Both panels represent models predicting wound healing between T2 (24 hours after wounding) and T3 (7 days after wounding).

In model 2, wound healing improved over time (*b* = −1.110, *t*_137_ = −8.907; *P* < .001). Main effects of oxytocin (*b* = −0.047, *t*_77_ = −1.007; *P* = .32) and affectionate touch (*b* < .001, *t*_57_ = 0.153; *P* = .88) were nonsignificant, as were all 2-way interactions (oxytocin × touch: *b* = 0.002, *P* = .69; time × touch: *b* = 0.004, *P* = .73; time × oxytocin: *b* = 0.314, *P* = .08). A significant 3-way interaction (time × oxytocin × affectionate touch) indicated that greater daily affectionate touch in the oxytocin group predicted improved wound healing (*b* = −0.038, *t*_137_ = −2.091; *P* = .04) (Figure 3A), which remained robust in sensitivity analyses (*b* = −0.037, *t*_135_ = −2.057; *P* = .04).

In model 3, wound healing again improved over time (*b* = −1.088, *t*_137_ = −12.540; *P* < .001). Main effects of oxytocin (*b* = −0.058, *t*_77_ = −1.704; *P* = .09) and sexual activity (*b* = 0.005, *t*_57_ = 0.394; *P* = .70) were nonsignificant, as were all 2-way interactions (oxytocin × sexual activity: *b* = 0.019, *P* = .30; time × sexual activity: *b* = 0.012, *P* = .79; time × oxytocin: *b* = 0.204, *P* = .12). A significant 3-way interaction (time × oxytocin × sexual activity) indicated that higher daily sexual activity in the oxytocin group predicted greater wound healing (*b* = −0.145, *t*_137_ = −2.122; *P* = .04) (Figure 3B). After sensitivity analysis, the effect was nonsignificant (*b* = −0.131, *t*_135_ = −1.900; *P* = .06) but directionally consistent (Table 1).

**Table 1.** Models Predicting Wound Healing^a^

Fixed effect	Model 1: wound healing by oxytocin and PAT				Model 2: wound healing by oxytocin and touch				Model 3: wound healing by oxytocin and sexual activity			
	Value (SE)	*df*	*t* Value	*P* value	Value (SE)	*df*	*t* Value	*P* value	Value (SE)	*df*	*t* Value	*P* value
Intercept (time zero)	1.676 (0.069)	286	24.128	<.001	1.407 (0.091)	137	15.440	<.001	1.412 (0.086)	137	16.364	<.001
Time (T1, T2, T3)^b^	−0.103 (0.031)	286	−3.278	.001	NA	NA	NA	NA	NA	NA	NA	NA
Time (T2, T3)^b^	NA	NA	NA	NA	−1.110 (0.125)	137	−8.907	<.001	−1.088 (0.087)	137	−12.540	<.001
Wound severity at T1^b^	NA	NA	NA	NA	0.045 (0.012)	57	3.646	.001	0.043 (0.012)	57	3.494	.001
Oxytocin	−0.021 (0.043)	76	−0.493	.62	−0.047 (0.046)	77	−1.007	.32	−0.058 (0.034)	77	−1.704	.09
PAT	0.037 (0.043)	76	0.860	.39	−0.009 (0.024)	77	−0.353	.73	−0.011 (0.024)	77	−0.460	.65
Sex	−0.069 (0.019)	67	−3.607	.001	−0.070 (0.022)	57	−3.217	.002	−0.070 (0.022)	57	−3.267	.002
Age	0.001 (0.002)	67	0.443	.66	0.001 (0.002)	57	0.609	.55	0.001 (0.002)	57	0.638	.53
Time × oxytocin	0.030 (0.044)	286	0.677	.50	0.314 (0.180)	137	1.751	.08	0.204 (0.129)	137	1.572	.12
Time × PAT	0.007 (0.044)	286	0.163	.87	NA	NA	NA	NA	NA	NA	NA	NA
Oxytocin × PAT	0.049 (0.061)	76	0.810	.42	NA	NA	NA	NA	NA	NA	NA	NA
Time × oxytocin × PAT	−0.125 (0.063)	286	−1.983	.048	NA	NA	NA	NA	NA	NA	NA	NA
Touch	NA	NA	NA	NA	0.000 (0.003)	57	0.153	.88	NA	NA	NA	NA
Time × touch	NA	NA	NA	NA	0.004 (0.012)	137	0.351	.73	NA	NA	NA	NA
Oxytocin × touch	NA	NA	NA	NA	0.002 (0.005)	57	0.406	.69	NA	NA	NA	NA
Time × oxytocin × touch	NA	NA	NA	NA	−0.038 (0.018)	137	−2.091	.04	NA	NA	NA	NA
Sexual activity	NA	NA	NA	NA	NA	NA	NA	NA	0.005 (0.012)	57	0.394	.70
Time × sexual activity	NA	NA	NA	NA	NA	NA	NA	NA	0.012 (0.045)	137	0.271	.79
Oxytocin × sexual activity	NA	NA	NA	NA	NA	NA	NA	NA	0.019 (0.018)	57	1.044	.30
Time × oxytocin × sexual activity	NA	NA	NA	NA	NA	NA	NA	NA	−0.145 (0.069)	137	−2.123	.04

Abbreviations: EMA, ecological momentary assessment; NA, not applicable; PAT, structured positive interaction (Partner Appreciation Task).

^a^ Model 1 tested the prediction of wound healing by oxytocin and PAT. Models 2 and 3 tested the prediction of wound healing based on oxytocin and affectionate touch during the EMA (model 2), and sexual activity during the EMA (model 3). Given that models 2 and 3 required the calculation of ecologically valid interactions, they are based on time points T2 (pre-EMA) and T3 (post-EMA), controlling by the wound severity of the first time point, T1.

^b^ Time T1 is 1 hour after wounding; T2, 24 hours after wounding; and T3, 7 days after wounding.

### Effects of Oxytocin Administration and Physical Intimacy on Psychobiological Stress Responses

Exploratory lagged models indicated that oxytocin administration did not significantly affect the number of affectionate touches (*b* = −0.066, *z* = −0.354, *P* = .72) or sexual activities (*b* = 0.100, *z* = 0.303, *P* = .76). Higher perceived relaxation was positively associated with subsequent engagement in affectionate touch (*b* = 0.253, *z* = 3.696, *P* < .001) and sexual activity (*b* = 0.605, *z* = 3.917, *P* < .001), suggesting that lower perceived stress precedes increases in physical intimacy (Table 2).

**Table 2.** Perceived Stress, Cortisol, and Intimate Interaction Models^a^

Fixed effect	Model 4: touch predicted by oxytocin and stress				Model 5: sexual activity predicted by oxytocin and stress				Model 6: AUCg by oxytocin and touch				Model 7: AUCg by oxytocin and sexual activity			
	Value (SE)	*df*	*z* Value	*P* value	Value (SE)	*df*	*z* Value	*P* value	Value (SE)	*df*	*z* Value	*P* value	Value (SE)	*df*	*z* Value	*P* value
Intercept	−5.429 (0.723)	NA	−7.510	<.001	−2.521 (1.008)	NA	−2.501	.01	8.395 (0.381)	488	22.041	<.001	8.404 (0.380)	488	22.130	<.001
Time point EMA	0.908 (0.086)	NA	10.567	<.001	0.212 (0.158)	NA	1.338	.18	NA	NA	NA	NA	NA	NA	NA	NA
Oxytocin (daily)	−0.066 (0.186)	NA	−0.354	.72	0.100 (0.329)	NA	0.303	.76	NA	NA	NA	NA	NA	NA	NA	NA
Stress (within participant)	0.253 (0.069)	NA	3.696	<.001	0.605 (0.155)	NA	3.917	<.001	NA	NA	NA	NA	NA	NA	NA	NA
Sex	0.308 (0.224)	NA	1.378	.17	−0.026 (0.254)	NA	−0.102	.92	0.124 (0.062)	63	1.997	.05	0.115 (0.062)	63	1.853	.07
Age	0.017 (0.022)	NA	0.769	.44	−0.063 (0.028)	NA	−2.230	.03	−0.008 (0.006)	63	−1.236	.22	−0.008 (0.006)	63	−1.258	.21
Oxytocin (group assignment)	NA	NA	NA	NA	NA	NA	NA	NA	0.085 (0.072)	78	1.188	.24	0.089 (0.071)	78	1.267	.21
Day	NA	NA	NA	NA	NA	NA	NA	NA	−0.041 (0.010)	488	−4.188	<.001	−0.038 (0.010)	488	−3.893	<.001
BMI	NA	NA	NA	NA	NA	NA	NA	NA	0.018 (0.014)	63	1.316	.19	0.017 (0.014)	63	1.249	.22
Physical activity	NA	NA	NA	NA	NA	NA	NA	NA	0.016 (0.019)	488	0.836	.40	0.021 (0.019)	488	1.101	.27
Liquid intake	NA	NA	NA	NA	NA	NA	NA	NA	0.019 (0.024)	488	0.802	.42	0.016 (0.024)	488	0.654	.51
Food intake	NA	NA	NA	NA	NA	NA	NA	NA	−0.011 (0.025)	488	−0.464	.64	−0.013 (0.024)	488	−0.526	.60
Affectionate touch	NA	NA	NA	NA	NA	NA	NA	NA	−0.021 (0.013)	488	−1.586	.11	NA	NA	NA	NA
Sexual activity	NA	NA	NA	NA	NA	NA	NA	NA	NA	NA	NA	NA	−0.083 (0.030)	488	−2.813	.005

Abbreviations: AUCg, area under the curve with respect to the ground; BMI, body mass index; EMA, ecological momentary assessment; NA, not applicable; PAT, structured positive interaction (Partner Appreciation Task).

^a^ This table presents results of hierarchical linear models. Models 4 and 5 examined how perceived stress (here low levels represent high stress) and oxytocin predict subsequent changes in the likelihood of affectionate touch (model 4), and sexual activity (model 5). Models 6 and 7 tested whether oxytocin, affectionate touch, and sexual activities predict daily cortisol secretion, measured as AUCg. Because of estimation issues and low variance at the couple level, models 4 and 5 included observations nested in individuals. Models 6 and 7 included observations nested in individuals who were nested in couples.

Regarding aggregated daily cortisol secretion (AUCg), oxytocin administration showed no main effect (*b* = 0.085, *t*_78_ = 1.188; *P* = .24). Significant predictors of AUCg included measurement day (*b* = −0.041, *t*_488_ = −4.188; *P* < .001) and sexual activities (*b* = −0.083, *t*_488_ = −2.813; *P* = .005). The number of affectionate touch sequences was not associated with AUCg (*b* = −0.021, *t*_488_ = −1.586; *P* = .11).

## Discussion

This double-blind, randomized, placebo-controlled trial tested whether intranasal oxytocin, instructed positive interaction (PAT), and naturally occurring intimacy influence wound healing. Oxytocin enhanced wound healing only in interaction with social behaviors, by tendency with PAT and significantly with affectionate touch and sexual activity, whereas oxytocin or PAT alone showed no effect. These findings suggest that oxytocin amplifies the benefits of intimacy rather than exerting direct effects.

Previous animal data on this topic are mixed, with oxytocin alone showing no effect on healing,^31^ but synergistic effects with social interaction in hamsters^29^ and with social housing in mice.^30^ Human evidence remains scarce, limited to 1 study linking endogenous oxytocin with partner communication and faster healing.^33^

Despite early enthusiasm in oxytocin administration studies, more recent reviews have highlighted that findings from intranasal oxytocin research are inconsistent and studies are often underpowered.^47,48,49^ Several large-scale replications have failed to reproduce key effects, such as the link between oxytocin and trust,^50^ and null results have been reported in both healthy and clinical populations.^51^ Given these limitations, researchers have increasingly called for a shift from testing general main effects of oxytocin toward examining interactions that consider individual and contextual factors.^48,52^ As summarized by Yao and Kendrick,^53^ oxytocin effects in romantic contexts vary depending on factors like relationship type and perceived partner characteristics; for example, oxytocin enhances partner attractiveness, especially when the partner is seen as trustworthy.

At the biological and mechanistic level, both positive couple interactions and oxytocin have been associated with improved immune functioning, suggesting that their interplay may contribute to enhanced wound healing through reductions in cortisol levels and modulation of immune factors.^54^ Following the salience hypothesis of oxytocin,^55^ effects should be particularly strong when oxytocin levels are high in situations positively attributed: an interaction that we tried to stimulate by combined oxytocin administration and behavioral instructions. Our results align with this framework, as we observed no main effects of oxytocin administration but significant effects only in interaction with well-established social behaviors, such as affectionate touch and sexual activity, and less so with the behavioral intervention, the PAT. However, when considered alongside the immune data from a subsample of this study,^32^ immune changes associated with oxytocin and PAT in the previous analysis did not mediate wound healing here.

Beyond healing, physical intimacy predicted lower stress and cortisol, with lagged analyses showing that relaxation increased subsequent touch and sexual activity. This supports EMA studies linking stress to reduced intimacy.^56,57^ In contrast, some research suggests that individuals may seek closeness during stress^58,59^ as a form of common dyadic coping.^60^ This pattern aligns with the attachment theory by Bowlby, which views touch as a regular effect of safety and belonging.^61,62^ Taken together, our findings indicate that physical intimacy is more likely under relaxed conditions, highlighting the need for context-sensitive research on how stress influences intimacy, particularly considering individual differences in attachment security.^63^

### Limitations

Limitations include that healing was assessed only at 24 hours and 7 days, limiting sensitivity. The oxytocin × PAT effect was not robust to case exclusion, PAT adherence was incomplete (68%), and task timing was not standardized. Beliefs about group assignment were only indirectly assessed, and intimacy measures lacked standardized definitions. Finally, the sample was restricted to young, healthy, heterosexual couples, limiting generalizability.

## Conclusions

This study found that oxytocin alone did not enhance wound healing, nor did the behavioral intervention PAT. However, when combined with the PAT (by tendency) or with everyday intimacy (with more robust effects), oxytocin was associated with faster recovery. These modest, context-dependent effects provide preliminary insights into the neuroendocrine mechanisms through which intimate relationships can improve health, underscoring the need for larger, more diverse trials. Data support the view that oxytocin functions as a social amplifier rather than a standalone therapeutic.

## Notes

Supplement 1. Trial protocol

Supplement 2. **eTable 1.** Sociodemographic characteristics of the sample **eTable 2.** Oxytocin vs. placebo group differences in relationship and personality characteristics, and outcome variables **eTable 3.** PAT vs. nPAT group differences in relationship and personality characteristics, and outcome variables **eFigure.** Exemplary illustration of wound photographs on day 1, day 2, and day 7

Supplement 3. Data sharing statement
