# Supplementary material for: Intranasal Oxytocin and Physical Intimacy for Dermatological Wound Healing and Neuroendocrine Stress: A Randomized Clinical Trial
Source: JAMA Psychiatry. 2025 Nov 12;83(2):118–27. doi: 10.1001/jamapsychiatry.2025.3705 (PMC12613093; doi:10.1001/jamapsychiatry.2025.3705)
Supplement: Supplement 1. — Trial protocol [file jamapsychiatry-e253705-s001.pdf]

Protocol of the planned investigation:

Oxytocin, couple interaction and wound healing

A placebo-controlled study in two substudies on the influence of the hormone oxytocin on couple interaction, wound healing and the perception of couple-specific stimuli

Dr phil. Beate Ditzen

lic. phil. Corinne Spörri

Prof. Dr rer. nat. Markus Heinrichs

Dr Severin Läuchli, MD

Prof. Dr rer. nat. Ulrike Ehlert

Date, signature (principal investigator)	Date, signature (sponsor)
Dr Severin Läuchli<br>University Hospital Zurich<br>Dermatological Clinic<br>Gloriastrasse 31<br>CH-8091 Zurich	Dr Beate Ditzen<br>Institute of Psychology<br>Department of Clinical Psychology and Psychotherapy<br>Binzmühlestr. 14/26<br>CH-8050 Zurich

CONFIDENTIAL

The information in this study protocol is strictly confidential and the property of the Psychological Institute of the University of Zurich and the Department of Dermatology of the University Hospital Zurich. It is intended for the information of the investigators, the other persons involved in the study, the ethics committees and the regulatory authorities. This study protocol may not be reproduced - in whole or in part - or passed on to third parties without the consent of Dr B. Ditzen and Dr S. Läuchli.

STUDY SYNOPSIS

**Sponsor-Investigator:**	Dr Beate Ditzen
**Title:**	Oxytocin, couple interaction and wound healing<br>Sub-study 1: Oxytocin, couple interaction and wound healing in everyday life<br>Sub-study 2: Eye-tracking of oxytocin and couple-specific behaviour
**Short description:**	Oxytocin, couple interaction and wound healing
**Protocol version and date:**	Version 6 from 03/02/2011
**Clinical phase:**	Clinical study phase
**Study design:**	Double-blind, randomised, placebo-controlled
**Duration of study:**	Sub-study 1: 1 week, Sub-study 2: 1 or 2 x approx. 2h
**Study centres**	Monocentric
**Investigator(s):**	Dr Beate Ditzen, Dr Severin Läuchli, Dr Markus Heinrichs, Dr Ulrike Ehlert
**Aims and objectives of the clinical trial**	Sub-study 1: Wound healing after one week, recorded via transepidermal water loss (TEWL), wound size and epitelisation of the wound<br>Sub-study 2: Interest in the images presented, recorded using gaze duration, gaze focus and subjective evaluation of the images
**Number of subjects:**	Sub-study 1: 200 (100 pairs)<br>Sub-study 2: 88
**Inclusion criteria**	Healthy test subjects
**Exclusion criteria**	Acute or chronic mental or physical illness, alcohol or smoking, pregnancy and breastfeeding in women
**Investigational product:**	Oxytocin (Syntocinon, manufacturer: Novartis Pharma AG, Switzerland)<br>Sub-study 1: 1 x 24 IU, 5 x 32 IU/day<br>Sub-study 2: 1 or 2 x 24 IU at intervals of approx. 2 weeks
**Duration of administration**	Sub-study 1: 6 days<br>Sub-study 2: Single or double administration
**Reference therapy,:**	none
**Timetable**	02/2011 until 02/2013
**Statistical analysis:**	Hierarchical linear models (HLM), analyses of variance (ANOVAs), regressions, correlations
**GCP Declaration:**	This study is conducted in compliance with the protocol, the current version of the Helsinki Declaration and ICH-GCP, as well as national guidelines and legislation

VISIT PLAN Sub-study 1:

Study periods	***Recruitment***	***Treatment period***					***Follow-up***
Visit		***1***	***2***	***3***	***4***	***5***	***6***
Day		***1***	***2***	***3***	***4***	***5***	***6***
Patient consent		*x*					
Demographic data		*x*					
Medical history/ concomitant diseases	*x*	*x*					
Inclusion/exclusion criteria	*x*	*x*					
Physical examination		*x*					*x*
Vital signs		*x*					*x*
Cytokine laboratory		*x*	*x*				
Randomisation		*x*					
Patient diary			*x*	*x*	*x*	*x*	*x*
Test product application		*x*	*x*	*x*	*x*	*x*	*x*
Primary variables		*x*	*x*				*x*
AEs and SAEs		*x*	*x*	*x*	*x*	*x*	*x*

VISIT PLAN Sub-study 2:

Study periods	***Recruitment***	***Treatment period***	
Visit		***1***	***2***
Day		***1***	***2***
Patient consent		*x*	*x*
Demographic data		*x*	
Medical history/ concomitant diseases	*x*	*x*	
Inclusion/exclusion criteria	*x*	*x*	
Physical examination		*x*	*x*
Vital signs		*x*	*x*
Randomisation		*x*	*x*
Test product application		*x*	*x*
Primary variables		*x*	*x*
AEs and SAEs		*x*	*x*

Table of contents

1 List of abbreviations ... 1
2 persons involved ... 2
3 Ethics ... 4
3.1 Ethics committee and official notification ... 4
3.2 GCP-compliant study conduct ... 4
3.3 Patient information and declaration of consent ... 4
4. introduction ... 6
4.1 Background and rationale of the clinical study ... 6
4.2 Current state of research ... 6
4.2.1 Couple interaction and health ... 7
4.2.2 Oxytocin, stress and couple interaction ... 7
4.2.3 Oxytocin, couple interaction and wound healing ... 9
4.3 Name and indication of the investigational medicinal product ... 9
4.4 Preclinical data ... 10
4.5 Clinical data ... 10
4.6 Dosing regimen of the investigational product ... 10
5 Study design and study procedure, sub-study 1 ... 11
5.1 Questions ... 11
5.2 Hypotheses ... 11
5.3 Main target parameters ... 11
5.4 Study design ... 11
5.4.1 Study sample & power analysis ... 11
5.4.2 Inclusion and exclusion criteria ... 12
5.4.3 Recruitment ... 13
5.4.4 Procedure ... 14
5.5 Psychological examination methods ... 17
5.5.1 Self-report data ... 17
5.5.2 Standardised instruction of positive pair interaction ... 18
5.6 Physiological examination methods ... 18
5.6.1 Intranasal application of oxytocin ... 18
5.6.2 Suction bubble application ... 21
5.6.3 Ecological Momentary Assessment (EMA) ... 22
5.7 Data collected and data analyses ... 23
5.7.1 Behavioural and self-report data ... 23
5.7.2 Biological data ... 23
5.7.3 Statistical data analyses ... 24
6 Study design and study procedure, sub-study 2 ... 25
6.1 Research question ... 25
6.2 Hypotheses ... 25
6.3 Main target parameters ... 25
6.4 Study design ... 25
6.4.1 Study sample and power analyses ... 26
6.4.2 Inclusion and exclusion criteria ... 26
6.4.3 Procedure of the experiment ... 27
6.5 Research methods ... 29
6.5.1 Psychological examination methods ... 29
6.5.2 Physiological examination methods: Oxytocin application ... 29
6.6 Collected data and data analyses ... 31
6.6.1 Behavioural and self-report data ... 31
6.6.2 Statistical data analyses ... 31
7 Risk-benefits ratio and ethical considerations ... 32
7.1 Ethical aspects ... 32
7.2 Confidentiality of respondent data ... 32
7.3 Potential risks and protection against risks ... 33
7.3.1 Syntocinon (oxytocin) nasal spray ... 33
7.3.2 Suction blister application, measurement of wound fluid and measurement of the wound ... 33
7.3.3 For women of childbearing age ... 34

7.3.4 For men ... 34
7.3.5 Measurements in everyday life ... 34
7.3.6 Stimulus material ... 35
7.3.7 Eye tracking ... 35
7.3.8 Psychological questionnaires ... 35
7.4 Efficacy and safety variables ... 35
7.5 (Serious) adverse events, side effects ... 36
7.5.1Adverse event (AE) ... 36
7.5.2 Adverse drug reaction (ADR) ... 36
7.5.3 Serious Adverse Event (SAE) ... 37
7.5.4 Suspected Serious Unexpected Adverse Reaction (SUSAR) ... 37
7.6 Documentation of (S)AEs ... 37
7.7 Assessment of (Serious) Adverse Events ... 38
7.8 Reporting of serious adverse events (SAEs) ... 39
7.9 Follow-up of (serious) adverse events ... 41
8 Data Quality Assurance ... 41
8.1 Monitoring ... 41
8.2 Audits and inspections ... 42
8.3 Specification of source documents ... 42
8.4 Documentation and storage of data ... 43
8.5 Data protection and confidentiality ... 44
9 Insurance ... 44
10 Study registration ... 45
11 Publications ... 45
12 Timetable ... 45
13 signatures ... 46
14 References ... 47

1 List of abbreviations

AE	Adverse event
CRF	Case Report Form
GCP	Good Clinical Practice
GMP	Good Manufacturing Practice
I	International Conference on Harmonisation
IB	Investigator's Brochure
ISF	Investigator Site File (study centre folder)
PI	Principal Investigator (principal investigator)
SAE	Serious Adverse Event
SDV	Source Data Verification (original data synchronisation)
SOP	Standard Operating Procedure
SUSAR	Suspected Unexpected Serious Adverse Reaction (suspected case of an unexpected serious adverse reaction)
TMF	Trial Master File (central trial folder)
KAZ	Cantonal Pharmacy Zurich

2 persons involved

Sponsor-Investigator<br>(Principal Investigator):	**Investigator:**<br>Name: Dr med. Severin<br>Address: University Hospital Zurich,<br>Dermatological Clinic, Gloriastr. 31, CH-8091 Zurich<br>Email: Severin.Laeuchli@usz.ch<br>Tel: +41 44 255 28 11<br>Fax: +41 44 255 44 03<br><br>**Sponsor:**<br>Name: Dr phil. Beate Ditzen<br>Address: University of Zurich, Department of Psychology, Clinical Psychology and Psychotherapy, Binzmühlestr. 14/ Box 26, CH-8050 Zurich<br>Email: b.ditzen @psychologie.uzh.ch<br>Tel: +41 44 635 7365<br>Fax: +41 44 635 7359
Study centre(s):	Sub-study 1:<br>University Hospital Zurich,<br>Dermatological Clinic<br>Address: Gloriastr. 31, CH-8091 Zurich<br><br>Sub-study 2:<br>University of Zurich<br>Institute of Psychology<br>Address: Binzmühlestr. 14/ Box 26
Study Coordinator:	Name: lic. phil. Corinne Spörri<br>Address: University of Zurich, Department of Psychology, Clinical Psychology and Psychotherapy, Binzmühlestr. 14/ Box 26, CH-8050 Zurich<br>Email: c.spoerri@psychologie.uzh.ch<br>Tel: +41 44 635 7369<br>Fax: +41 44 635 7359
Investigator(s):	Name: Prof Dr Markus Heinrichs<br>Address: Albert-Ludwigs-University of Freiburg,<br>Institute of Psychology, Biological and Differential Psychology, Stefan-Meier-Strasse 8 D-79104 Freiburg i. Br., Germany<br>Email: heinrichs@psychologie.uni-freiburg.de<br>Tel: +49-761-203-3024<br>Fax: +49-761-203-3023<br><br>Name: Prof. Dr Ulrike Ehlert<br>Address: University of Zurich, Department of Psychology, Clinical Psychology and Psychotherapy, Binzmühlestr. 14/ Box 26, CH-8050 Zurich<br>Email: u.ehlert@psychologie.uzh.ch<br>Tel: +41 44 635 73 50<br>Fax: +41 44 635 7359
Laboratory	Name: Adrian Urwyler<br>Address: CYTOLAB<br>Email: a.urwyler@gmx.ch<br>Tel: +41 078 612 61 39<br>Fax: -
Cantonal pharmacy (KAZ)	Name: Theodor Sonderegger<br>Address: Head of Galenics, Zurich Cantonal Pharmacy

	Spöndlistrasse 9, 8006 Zurich<br>Email: theodor.sonderegger@kaz.zh.ch<br>Tel: +41 44 255 62 16<br>Fax: +41 44 255 91 17
Centralised data management	Name: Dr Andreas U. Freiburghaus<br>Address: Head of Data Management, Clinical Trials Centre - Centre for Clinical Research ZKF,<br>University Hospital and University of Zurich,<br>Rämistrasse 100, CH - 8091 Zurich<br>Email: andreas.freiburghaus@usz.ch<br>Tel: +41 44 634 56 61<br>Fax: -

3 Ethics

3.1 Ethics committee and official notification

In accordance with the Swiss Therapeutic Products Act, the clinical trial will not be started until the favourable assessment of the ethics committee (EC) responsible for the principal investigator (PI) and the approval of the competent authority (Swissmedic/FOPH) have been obtained. Changes to the study protocol must also be authorised by these institutions.

The decision of the responsible ethics committee and Swissmedic to conduct this study will be sent to the sponsor-investigator in writing.

3.2 GCP-compliant study conduct

This study will be conducted in compliance with the protocol, the Helsinki Declaration and according to Good Clinical Practice (GCP), as well as Swiss guidelines and legislation.

The responsible EC and authorities are informed about the progress of the study and the end/stop of the study in accordance with local requirements by means of an annual safety report and interim reports.

Under certain conditions, the sponsor-investigator may terminate the clinical trial prematurely due to

- ethical concerns,
- insufficient patient recruitment,
- if the safety or benefit of the participating persons is doubtful or jeopardised,
- Change in accepted clinical practice, whereupon continuation of the study would be unwise,
- Achieving a positive or negative result earlier than expected.

3.3 Patient information and declaration of consent

Before the start of the study, each person participating in the study (or their legal representative) must give their written consent to the investigator after having been fully informed in oral and written form about the nature, significance and scope of the clinical study in a way that they can understand. The content of this information will be documented on the informed

consent form. The participant will be informed if significant new findings about the investigational medicinal product arise during the study. The participant is informed that participation is voluntary and that they can withdraw their participation at any time without affecting their further medical care.

The participating person is informed that their study data/medical records can be viewed by authorised persons and authorities.

The declaration of consent to participate in the clinical trial is dated and signed by the participant and the investigator. One copy of the signed patient information/declaration of consent is given to the participating person, the second copy is filed in the trial centre folder.

The patient information and declaration of consent will be submitted together with the protocol to the responsible EC for review and approval. It is expressly pointed out that no examinations may be carried out in connection with the study until a legally valid declaration of consent has been submitted.

4. Introduction

This document is the protocol for conducting a clinical study in humans. This study is conducted in compliance with the protocol, the current version of the Helsinki Declaration and ICH-GCP, as well as national guidelines and legislation.

4.1 Background and rationale of the clinical study

The planned research project aims to answer the question of whether the hypothalamus hormone oxytocin modulates the perception of couple-specific stimuli (such as bonding and sexuality) and couple interaction. The central question to be investigated is whether oxytocin in interaction with couple interaction has an influence on wound healing and thus on individual health. It is assumed that the assumed effect is determined by the perception of couple-specific variables and is therefore specific to the interaction of couples. This specificity is to be tested in a sub-study. The project therefore consists of two parts:

1) In sub-study 1, the interaction of oxytocin and couple interaction in the everyday life of couples on the healing of a standardised applied wound is investigated. In a 2x2 design involving 100 couples, the influence of positive couple interaction and oxytocin, or of no instructed interaction and placebo, on wound healing over the course of a week is tested. It is assumed that oxytocin and positive couple interaction have a positive influence on wound healing. It is also assumed that oxytocin and positive couple interaction have a positive influence on the couples' mood in everyday life and reduce everyday stress.

2) Sub-study 2 of the project will specifically investigate the influence of oxytocin on the perception and evaluation of attachment stimuli and sexual stimuli compared to neutral control stimuli. This study will investigate the interest in the stimuli in 88 people by questioning the test subjects on the one hand and by means of eye tracking on the other.

4.2 Current state of research

Involvement in close social relationships has significant consequences for an individual's health and even survival (Berkman, Glass, Brissette & Seeman, 2000). Epidemiological studies have repeatedly shown that social isolation increases the risk of ill health - to an extent comparable to the risk of smoking (House, Landis & Umberson, 1988). Social support has therefore also been defined by the WHO as one of the most important sources of mental and physical health (Wilkinson & Marmot, 2003). Marriage, as one of the most common forms of

relationship in adulthood, can be interpreted as a continuing source of social support, and it has been shown that the chances of survival after medical illness increase significantly for happily married people. In turn, however, repeated or chronic stress (e.g. through repeated couple conflicts) continuously strains the physical stress systems, such as the hypothalamic-pituitary-adrenal axis (HHNA) and the autonomic nervous system (ANS), and thus impairs health (Ditzen & Heinrichs, 2007; Kiecolt-Glaser & Newton, 2001; Robles & Kiecolt-Glaser, 2003). These findings are consistent with those that have established a link between chronic changes in the endocrine stress system and mortality and morbidity (Gruenewald et al., 2006; Mc Ewen, 1998).

4.2.1 Couple interaction and health

It is therefore assumed that stable relationships lower stress levels and thus reduce the individual risk of illness (Kiecolt-Glaser, 2005; Burman & Margolin, 1992). However, the quality of the relationship also plays a major role here: people in an unhappy relationship are confronted with a similar health risk as those living alone (Coyne et al., 2001). In addition, unhappy couples show an increased autonomic and endocrine stress response (Ewart et al., 1991; Malarkey et al., 1994) as well as reduced immune regulation (Kiecolt-Glaser et al., 1993) compared to happy couples.

In rodents, touching leads to reduced stress responses and the promotion of wound healing (Glasper & DeVries, 2005), a decrease in corticosterone and an increase in glucocorticoid receptor gene expression in the hippocampus, cortex and midbrain (Jutapakdeegul et al., 2003) and reduced blood pressure (Holst et al., 2002). Humans also show reduced neuronal activity in stress-associated brain regions when their partner holds their hand (Coan et al., 2006), and even 10 minutes of handholding reduces cardiovascular activity in response to stress (Grewen et al., 2003). Even more specifically in relation to touch, we were able to show (Ditzen et al. 2007) that positive touch by the partner significantly reduced salivary cortisol and heart rate during laboratory stress, while purely verbal support did not result in reduced stress responses. The self-reported tenderness of couples in everyday life is also associated with reduced salivary cortisol levels (Ditzen, Hoppmann et al., 2008).

4.2.2 Oxytocin, stress and couple interaction

The nonapeptide oxytocin is produced in the paraventricular and supraoptic nucleus of the hypothalamus, from where oxytocinergic neurons project into the pituitary gland and subse-

quently release oxytocin into the blood. At the same time, oxytocin plays an important role as a neurotransmitter in the brain. It is known from animal studies that the peptide plays a prominent role in the control of social bonding behavior (e.g. mother-infant bonding, pair bonding; Carter, 1998; Insel & Young, 2001) and appears to modulate numerous forms of positive approach behavior in mammals, in addition to its known importance for birth and breastfeeding. In addition to these prosocial effects, the administration of oxytocin directly into the ventricular system of the brain reduces anxiety and stress response in rodents (Windle, Shanks, Lightman & Ingram, 1997). Oxytocin could therefore also be a central mechanism in humans that mediates the effect of social relationships on the physical stress systems in the central nervous system. Direct effects of oxytocin on behavior have only recently been investigated, as only a very small amount of intravenously injected oxytocin crosses the blood-brain barrier and can therefore hardly exert any behavioral effects in the brain. Since it has been shown that intranasally administered neuropeptides apparently cause central nervous effects (Born et al., 2002), an effect of oxytocin nasal spray on social behavior and specifically on social support has also been investigated in humans in several studies (Heinrichs & Gaab, 2007). For example, intranasal oxytocin administration increased the buffering effects of social interaction on the psychoneuroendocrine stress response in non-human primates (Parker et al., 2005) and in humans (Heinrichs et al., 2003). These effects appear to be mediated by reduced activity of the amygdala (Domes, Heinrichs, Glascher et al., 2007; Kirsch et al., 2005). Particularly interesting in this context are studies that show that oxytocin modulates the formation and maintenance of social relationships in rodents.

In humans, Ditzen et al (2009) recently showed that intranasally administered oxytocin increases the duration of positive behavior in couples compared to negative behavior during couple conflict and reduces salivary cortisol in both partners. These results suggest that oxytocinergic mechanisms may influence health in couple relationships through altered couple interaction on behavior and a reduced physical stress response. This effect may be mediated by the increased availability of positive relationship memories (Guastella, Mitchell & Mathews, 2008; Heinrichs et al., 2004), increased trust (Kosfeld et al., 2005) and reduced anxiety (Domes, Heinrichs, Glascher et al., 2007; Kirsch et al., 2005) during couple interactions.

It remains to be investigated whether these results are relevant for individual immune capacity and health, which central activation patterns mediate this effect, and whether these effects are

specific to couple relationships and attachment relationships compared to non-social situations.

4.2.3 Oxytocin, couple interaction and wound healing

Wound healing can be interpreted as a sign of healthy immune function and thus of health in general. It involves three phases: 1) the inflammation phase, in which cytokine levels (interleukins 1 and 6: IL-1, IL-6), tumor necrosis factor α (TNF-α), and IL-8 increase in the wounded tissue, 2) the proliferation phase, which is characterized by the recruitment and replication of cells necessary for tissue and capillary regeneration, and 3) the epithelialization phase with collagen synthesis (Clark, 1996).

In addition to various factors that influence wound healing, such as age, gender, vitamin and trace element deficiencies (vitamin C, vitamin A, zinc) and systemic diseases (e.g. diabetes) (see Clark, 1996), there is evidence that chronic stress inhibits wound healing (for a review see Christian et al, 2006; DeVries et al., 2007) by slowing the production of pro-inflammatory cytokines (e.g. Il-1β and IL-6) in the wound through increased glucocorticoid (cortisol) concentrations (Hubner et al., 1996; Padgett et al., 1998). For example, Kiecolt-Glaser et al. (2005) found that negative pair interaction, as opposed to social support, slows wound healing in the laboratory. Interestingly, the group around DeVries (Detillion et al., 2004) was able to show in various studies with hamsters that social isolation causes an increased cortisol concentration and that treatment with oxytocin can in turn reduce cortisol and accelerate wound healing. This effect has not yet been investigated in humans.

4.3 Name and indication of the investigational medicinal product

In the planned project, the preparation "Syntocinon"® (manufacturer: Novartis Pharma, Basel, Switzerland), which contains the active ingredient oxytocin, will be used. Syntocinon is a commercially available drug that is used in obstetrics to induce labor before birth and to trigger the onset of lactation after birth. In the present study, the drug is used to mimic the mechanism of action of endogenous oxytocin in humans and to investigate it in a placebo-controlled manner.

4.4 Preclinical data

Syntocinon is a drug that is currently authorized for the Swiss market in a high-risk group (pregnant women and postpartum mothers). Preclinical and clinical data on the drug can be found in the drug brochure in the appendix.

4.5 Clinical data

The clinical data on the drug can be found in the drug brochure in the appendix.

4.6 Dosing regimen of the investigational product

In the planned project, Syntocinon and Syntocinon placebo will be used in healthy men and women to investigate the behavioral effects of naturally occurring oxytocin mechanisms in humans. The drug and dosage correspond to published studies in humans in which no side effects of the substance were reported (Heinrichs et al., 2003; Kosfeld et al., 2005, Ditzen et al., 2009, Domes et al., 2007). In sub study 1, the subjects receive 24 IU of oxytocin/placebo intranasally on the first day and 32 IU on each of the following 5 days. In sub-study 2, the test subjects receive 24 IU of oxytocin/placebo intranasally per laboratory test.

Syntocinon® is used in doses other than those described in the clinical application. However, the dosage corresponds to the information from previous studies. The Syntocinon concentration in sub-study 1 lies between the published quantities of 24 IU (Heinrichs et al., 2003, Kosfeld et al., 2005, Domes et al., 2007) and 40 IU (Ditzen, 2009), for which no side effects were reported. The concentration of 24 IU Syntocinon, which was administered intranasally at two measurements in sub-study 2, also corresponds to the published quantities.

5 Study design and study procedure, sub-study 1

Title: Oxytocin, couple interaction and wound healing in everyday life

5.1 Questions

Sub-study 1 investigates the influence of oxytocin vs. placebo on the instructed positive pair interaction, on parameters of the HHNA (salivary cortisol) and the ANS (salivary alpha amylase) and furthermore on the healing of a standardized suction blister wound.

5.2 Hypotheses

We hypothesise ***1)*** that, compared to placebo, oxytocin a) enhances instructed positive couple interaction, b) reduces the activity of psychobiological stress parameters and c) accelerates wound healing. In further exploratory hypotheses, the mediating influence of partnership quality and the couples' everyday interactions on these effects will be investigated.

5.3 Main target parameters

The main target parameter in sub-study 1 is the healing process, i.e. the reduction in size of a standardized suction blister wound on the non-dominant forearm one week after application. Furthermore, the immediate and delayed immune response to the application of the wound is recorded locally and systemically.

5.4 Study design

Sub-study 1 will be conducted according to ICH-GCP and in a randomized double-blind design. A total of 4 study arms will be investigated, which differ as follows: Oxytocin + positive couple interaction, oxytocin + no instructed interaction, placebo + positive couple interaction, placebo + no instructed interaction. 25 pairs per study arm will be randomly assigned. The probability of allocation to one of the 4 study arms is 25%, and the probability of allocation to oxytocin or placebo is 50%.

5.4.1 Research sample & power analysis

100 heterosexual couples aged 25-45 years will be analyzed. Using G*Power (Buchner, Faul, & Erdfelder, 1998), a sample size of N=76 was calculated for sub-study 1 with regard to the

main outcome variable "wound size" (see below) (effect f =.40; power = .82, alpha = .05). The total N is set to N=100 in order to be able to compensate for any dropouts.

5.4.2 Inclusion and exclusion criteria

The subjects must fulfil the following *inclusion criteria:*

- Heterosexual couple
- Between 25 and 45 years old
- In a stable relationship for at least one year (maximum 15 years) and living together
- No children
- Willingness to attend three laboratory appointments and carry out independent measurements at home
- Be healthy, e.g. have no acute, chronic physical, neurological, psychiatric or somatic illnesses
- Be a non-smoker (up to 5 cigarettes per day)
- fluent German
- Women: have a regular menstrual cycle (between 24 - 35 days)

Exclusion criteria

- Indications that the participant is unlikely to comply with the test plan (e.g. unwillingness to cooperate)
- Participation in another clinical trial within the last 4 weeks prior to inclusion
- Skin disease
- Accident with neurological sequelae
- Allergies or a history of known hypersensitivity to one of the drugs used or their ingredients, or to drugs with a similar chemical structure
- UV-B irradiation in the last 3 months before the examination
- Taking medication
- Drug use, addiction or other illnesses that do not allow the person concerned to the nature, scope and possible consequences of the clinical trial
- Overweight or underweight
- Daily alcohol consumption (> 60g alcohol, equivalent to approx. 7.5 dl wine or 2 litres beer per day)
- No participation of the Investigator or his/her family members, employees or other dependent persons
- Women: Pregnancy (for instructions on the double barrier to prevent pregnancy, see subject information) or intention to become pregnant during the course of the study

- Women: Breastfeeding

These criteria are queried with the interested participants in a telephone interview. No personal details are recorded during this telephone interview, so that no personal data can be linked to the inclusion or exclusion criteria before the consent form is signed. An appointment for the study is arranged with interested and suitable test subjects, at which the inclusion and exclusion criteria are discussed again and the test subjects sign the declaration of consent.

For this study, it is essential that certain things are controlled, as these activities or factors influence the measured values and may make them unusable. For this reason, the test subjects were asked to follow the instructions bellow:

- Do not drink coffee or other caffeinated drinks (cola, energy drinks, etc.), black tea or alcohol on the afternoon of the first laboratory appointment (approx. from 3 pm),
- Do not engage in excessive sport on the day of the examination,
- Do not smoke immediately before the 1st laboratory appointment,
- No physical exertion (cycling etc.) immediately before the 1st laboratory appointment,
- Do not eat anything heavy immediately before the 1st lab appointment.

5.4.3 Recruitment

The test subjects are recruited via circular mail from the mailing list for the recruitment of test subjects of the Psychological Institute of the University of Zurich, the description of the study in public media (ronorp, 20Minuten, NZZ), notices and websites for the recruitment of test subjects and receive CHF 500 per couple as compensation for participation. All couples will receive individual feedback on their results. On request, couples will be given the option of psychological counselling at the Outpatient Clinic for Cognitive Behavioral Therapy and Behavioral Medicine at the University of Zurich.

- Subjects may terminate their participation in the study at any time without giving reasons and without any disadvantage.
- The principal investigator may terminate a subject's participation in the study after weighing up the risk/benefit ratio. Possible reasons are
  - Health problems and adverse events
  - Refusal to use the nasal spray regularly

- Reasons for stopping the nasal spray immediately include

- Pregnancy
- Hypersensitivity to oxytocin or an excipient of the nasal spray

- Participation in the study must be terminated in the following cases:
  - If it subsequently transpires that the required inclusion criteria do not apply or at least one exclusion criterion applies
  - Cancellation of the investigation

5.4.4 Procedure

After a telephone interview in which inclusion and exclusion criteria are clarified, the first laboratory appointment is arranged with interested test subjects. After reading the information and signing the written declaration of consent at the first appointment, the participants receive a participant code and are given an individualized link to the study website. This link will take the subjects to a survey including questionnaires on personality, relationship quality, and chronic stress, which they can complete online. The online presentation of the questionnaires is organized via "Unipark" (www.unipark.com), a program for individualized scientific online data collection. Access to the electronic program "Unipark" and thus to the data of the participant is password-protected, and only the study coordinator has access. "Unipark" was specially designed by Globalpark AG for online research and administration of research data sets. The use of the program requires a license. The electronically collected data are only relevant in connection with the data collected in the laboratory. The electronic and laboratory data are merged using the respondent code, which the respondents receive at the first laboratory appointment and enter at the start of the online questionnaire. The merging of the electronic and laboratory data only takes place after the written declaration of consent, which the test subjects date and sign in the laboratory. If this is not done or the experiment is cancelled, the electronic data will be destroyed immediately. The data on the handheld computer cannot be viewed directly and is sent to a password-protected e-mail address and managed from there. Only the study management has access to this e-mail address.

1st laboratory appointment:

The first laboratory appointment takes place at the Dermatological Clinic of the University Hospital Zurich in the evening from 5 pm.

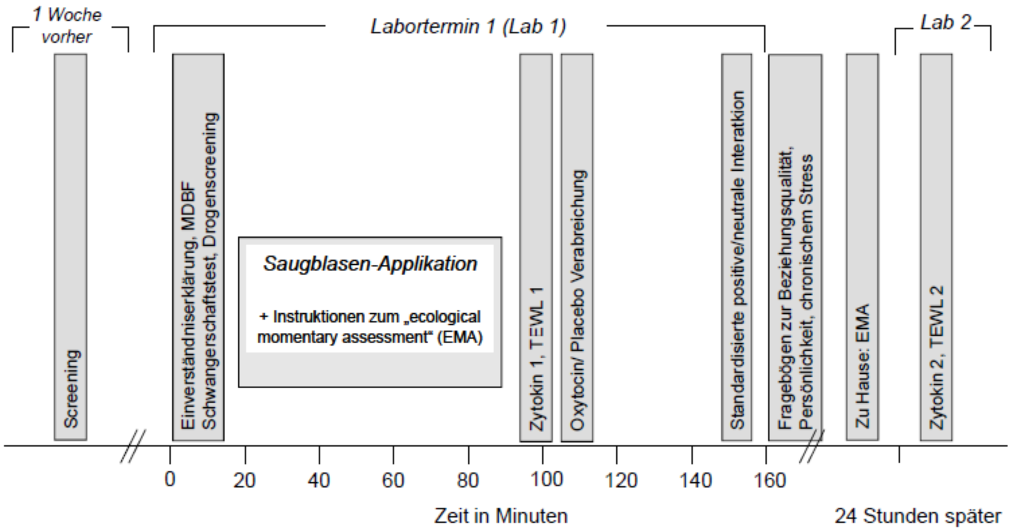

Figure1 : Flow chart, 1st + 2nd laboratory appointment

Study information and declaration of consent:

Before the start of the study, the study participants are once again informed about the procedure and aim of the study on the basis of the subject information. It is also explained to them that pregnancy excludes participation.

Admission procedure:

Study participants are asked to give their voluntary consent in writing. The investigator may only begin the study-specific examinations once the declaration of consent has been received. The enrolment examination is carried out according to the following criteria:

- Assignment of a subject number (in ascending order)
- Review of the inclusion and exclusion criteria
- Multi-drug test (urine test)
- For women: last menstruation + pregnancy test (standard urine test)

Subject code

The respondents are assigned an individual code, which they are asked to enter later at the beginning of the online questionnaire.

Suction bubble application

If there are no objections to the subjects' participation, all inclusion criteria are met and there are no exclusion criteria, they are asked how they feel. A doctoral student in dermatology then applies standardized suction blister wounds to the forearm under the supervision of the investigator Dr Severin Läuchli (both partners receive the suction blisters). During this procedure, the couples are informed about the second laboratory appointment, the momentary assessment part of the study, and the positive/negative couple interaction. Mood and pain perception are repeatedly recorded.

Oxytocin/placebo administration + interaction

After application of the wound, cytokine measurement 1 is performed and TEWL is measured. The test subjects are then given intranasal double-blind placebo or oxytocin (24IU, 3 sprays per nostril; both partners receive either placebo or oxytocin). The positive couple interaction is then explained and practiced. During this exercise, the couples are recorded on video. The first session lasts approximately 2.5 hours (see Figure 1).

Ecological Momentary Assessment (EMA):

For five days after the first laboratory appointment, the subjects are asked to chew saliva vials at 6 times/day to determine salivary cortisol and alpha-amylase levels (see Fig. 2). Four times a day, they were asked about stress, affect, and social interactions using a handheld computer. The confidentiality of the data on the handheld computer is guaranteed, as the data cannot be read directly on the device and is sent to a password-protected e-mail address to which only the study management has access. During laboratory session 1, the couples are shown how to take the nasal spray independently during the EMA and how to carry out the instructed couple interaction as well as the saliva and well-being measurements at home following a signal from the handheld computer. The saliva and well-being measurements are carried out at home on all 5 days, while the pair interactions should only take place on 3 evenings, at the couple's discretion.

2nd laboratory appointment:

24 hours after the first laboratory appointment, the couples visit the Dermatology Clinic of the University Hospital for a second time and wound fluid is taken again by the medical doctoral student under the supervision of Dr Severin Läuchli for analysis of cytokines and TEWL.

3rd laboratory appointment:

During the third laboratory appointment, the size of the wound is determined non-invasively. This appointment again takes place at the dermatology clinic. Afterwards, the couples receive a debriefing and the expense allowance (see Fig. 2).

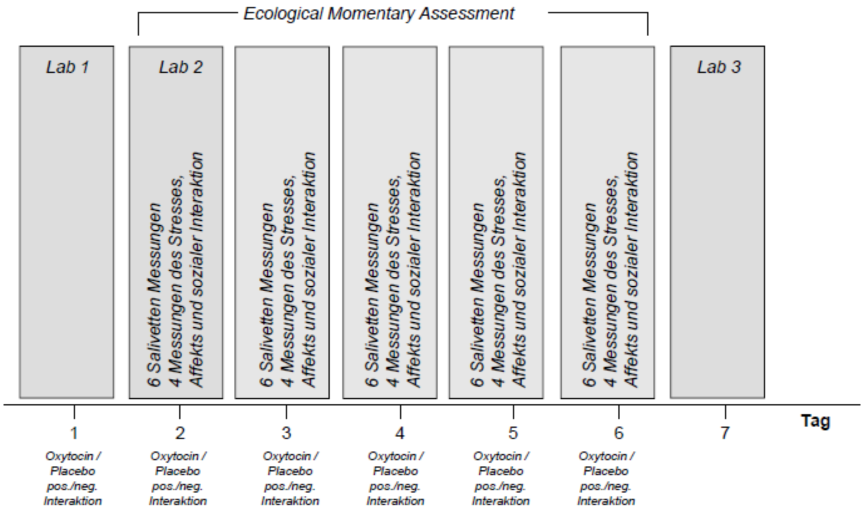

Figure2 : Overview of the entire study program.

5.5 Psychological examination methods

5.5.1 Self-report data

Self-assessed health (General Health Questionnaire, GHQ-12, Banks, 1983), personal information (standardized anamnesis questionnaire on housing and living situation, socio-economic status, etc.), partnership quality (partnership questionnaire, PFB, Hahlweg, 1996), dyadic coping (Dyadic Coping Inventory, DCI, Bodenmann, 2008), social support (Dyadic Coping Inventory, Bodenmann, 2008), and social support (Dyadic Coping Inventory, Bodenmann, 2008).), relationship quality (Partnership Questionnaire, PFB, Hahlweg, 1996), dyadic coping (Dyadic Coping Inventory, DCI, Bodenmann, 2008), social support (Berlin Social Support Scales, BSSS, Schwarzer & Schulze, 2000), personality traits (NEO-FFI, Borkenau & Ostendorf, 2008), chronic stress (Trier Inventory of Chronic Stress, TICS, Schulz, Schlotz & Becker, 2004), resilience (Resilience Scale, RS, Schumacher et al., 2004) and functional

somatic complaints (Screening for somatoform disorders 7T, SOMS-7T, Rief & Hiller, 1996) as well as functional somatic syndrome (FSSS, Gaab et al., 2003) are recorded online via personalized access after the first laboratory appointment.

During the laboratory appointments, mood (positive affect - negative affect, PANAS, Krohne et al., 1996) and pain (visual analogue scale) are repeatedly assessed.

5.5.2 Standardised instruction of pair interaction

During the first lab session, couples will either receive instructions for a standardized positive couple interaction or instructions to document a comparable time spent together during the coming week (without instructions on how to interact during this time).

50 couples are asked to rate 22 pre-defined positive or negative areas with regard to their relationship on a 4-dimensional Likert scale. They are also encouraged to add other areas not on the list. They are then asked which are the two most relevant positive or negative issues in their relationship and to discuss these. During this discussion, the couples are recorded on video. During three further, freely defined time points, the couples are asked to match the positive topics of the standard interaction to a recent interaction in everyday life. Couples in the control condition for this condition are asked to document the time they have spent together using the handheld computer.

5.6 Physiological examination methods

5.6.1 Intranasal application of oxytocin

In a double-blind and placebo-controlled design, participants will take oxytocin (Syntocinon, Novartis, Basel, Switzerland) or placebo intranasally at the first laboratory appointment and then twice daily for 5 days after the first laboratory appointment:

During the 1st laboratory appointment, the subjects take 24 IU (3 sprays in each nostril) before the instruction of the standardized positive interaction/no interaction.

On the day of the first laboratory appointment, the test subjects are asked to spray 2 bursts of oxytocin or placebo into each nostril at 2 times on the following 5 days (together with the saliva vial measurements 4 & 5) (a total of 2*2*4IU * 2/day= 32IU). This procedure is maintained on the following 5 measurement days at home. In total, the test subjects will therefore

ingest 1*24 and 5 * 32IU of either oxytocin or placebo (Ditzen, Schaer et al., 2009; Heinrichs et al., 2003; Kosfeld et al., 2005).

Compliance

The compliance of the test subjects with regard to taking medication at home is queried via the handheld computer. Non-compliance means that the respondent does not take the investigational product or does not state whether he/she has taken it and whether any side effects have occurred. The data of this person are excluded from the data analyses. The investigator checks the entries during the visit at the end of the week and takes back the empty packs or unused units.

Pre-treatment and concomitant therapy

No other treatments or medications are permitted during the study. Any concomitant therapies will be requested at the start of the study. All possible concomitant and/or emergency medication(s) must be documented in the CRF.

Packaging, labelling and delivery

The test preparation is given to the test subjects at home in the form of nasal sprays with a unique identification. The labels on the nasal sprays provide instructions on how often and in what dosage the nasal spray should be taken (see labels in the appendix). The study medication will be provided by KAZ and collected by the study coordinator before the start of the study. Upon receipt of the study medication/study material, an inventory is taken, a Drug Receipt Log is filled out and signed by the person who received the material. A thorough check is made to ensure that the consignment contains all the items on the delivery note. If the delivery contains defective or unusable study medication, this is recorded in the study documents. Regular drug reconciliation regarding allocation, administration and remaining study medication will be performed, documented with the Drug Reconciliation Form, and dated and signed by the study team. At the end of their participation in the study, the subjects return the nasal sprays, which are collected and documented by the study management. The returned nasal sprays will be kept until the end of data collection to enable any cross-checks with regard to blinding.

Blinding

The study is a placebo-controlled double-blind study. This means that the placebo contains all the same ingredients as the original medication - except for the active ingredient. The preparation of the placebo and blinding of the study medication is carried out by KAZ (GMP certificate see appendix). An assistant from the Chair of Clinical Psychology and Psychotherapy (lic. phil. Jana Campbell) who is not involved in the study will carry out the randomization using the "Research Randomizer" program and submit the randomization table to KAZ. The KAZ will hand over the table sealed in an envelope to the study management after the preparation of the test sample. This allows the investigator to unblind the code in an emergency. The code should only be broken in an emergency if the identity of the preparation must be known to the investigator in order to ensure the medical care of the participating person. After completion of data collection, the blinding is cancelled.

Storage conditions

The study medication is stored refrigerated in the KAZ. The unopened medication has a shelf life of three years. As the medication is not opened for blinding, all medication can be ordered and stored in one batch.

Return or destruction of the study medication

At the end of the study, a final review of the transport, dispensing and whereabouts of the study medication is carried out. This is documented with the Drug Reconciliation Form, dated and signed. Any discrepancies will be investigated, clarified and documented before any remaining study medication is destroyed. The destruction of the study medication is then noted in the study documents.

5.6.2 Suction bubble application

The suction blister method (Kiistala, 1968) is used in studies on cell and collagen synthesis in the skin in various diseases and after surgery (for a review, see Koivukangas, 2004), pro-inflammatory cytokines in wounds (Kuhns et al., 1992), and in studies investigating the effect of psychological predictors of wound healing (e.g. Kiecolt-Glaser et al., 2005). The method provides information on the inflammation and re-epithelialisation of wounds. A prolonged vacuum on the skin leads to detachment of the basement membrane at the level of the lamina lucida. When the blister is removed, the healing process of the basement membrane begins. Pro-inflammatory cytokines, trans epidermal water loss (TEWL), re-epithelialisation and wound size can be measured (cf. Jacobi et al., 2004) .

This study uses a suction blister protocol developed at the US National Institute of Health (NIH), which is a suitable and well-standardised in vivo model to assess the early and late wound healing process in humans (Kuhns et al., 1992; Enk et al., 1996): A plastic template is attached to the palm side on the surface of the non-dominant forearm and a vacuum is created using a pump until four small blisters form (1-1.5 hours). When the blisters have developed sufficiently, 1) the blister fluid formed from 2 blisters is carefully aspirated with a 1 cm^3 tuberculin syringe, 2) then the upper part of the blister (epidermis) is removed with a sterile knife. All blisters are treated with swabs and foil. Twenty-four hours later, the two remaining blisters are punctured. All blisters are treated with a hydrocoloid patch. The amount of the cytokines IL-1 and IL-6 is determined in the plasma. In order to control the variation of the

fluid volumes, the cytokine content of the fluids is calibrated as a function of the protein concentration (cf. Kuhns et al., 1992). The wound is measured after one week and compared with the adjacent non-wounded skin. The wound application is carried out by medical students under the supervision of Dr Severin Läuchli. The dependent variables of wound healing are the parameters 1) wound size, 2) trans-epidermal water loss (TEWL) and 3) epithelialisation. The cytokine content in the wound fluid is analyzed in terms of its influence as a mediating factor on wound healing.

5.6.3 Ecological Momentary Assessment (EMA)

The measurement of the psychological variables takes place on five days after the first laboratory appointment during four time points, the measurement of the saliva samples on six time points per day. The test subjects are contacted by a signal from the handheld computer (Palm Tungsten E2 handheld, or similar model) and asked to complete the questions on their current state of mind, stress and social interactions directly on the computer. The data is analysed using the freeware program "The Purdue Momentary Assessment Tool" (PMAT). The alarms are triggered at intervals of approximately three hours (Steptoe et al., 2008). In addition, the subjects are asked to perform 6 saliva measurements using salivettes (Salivette®, Sarstedt, Rommelsdorf, Germany) on all five days at the following times: immediately after waking up (measurement 1), 30 minutes after waking up (measurement 2), 2.5 hours after waking up (measurement 3), 8 hours after waking up (measurement 4), 12 hours after waking up (measurement 5) and before going to bed (measurement 6). Measurements 3-6 are compared with the four psychological measurements. The actual time of Salivette ingestion (and thus compliance with the study schedule) will be verified using electronic Aardex MEMS doses (Aardex Ltd, Zug, Switzerland). Drug accountability in sub-study 1 is ensured by asking the subjects at each measurement time point with the handheld computer whether they have taken the 2 sprays per nostril. In addition, the blinding table can be used to trace the batch number dispensed per subject. At the end of the study, the subjects must return the used nasal spray vials to the study coordinator so that the amount of nasal spray liquid actually taken can be verified. Compliance with Syntocinon® is recorded every day using a hand-held computer at and the intake of Syntocinon® is always recorded together with the saliva measurement, which is documented using the Aardex MEMS doses.

During the first laboratory appointment, the test subjects are explained how to use the EMA materials over the next five days. They also receive a written summary containing all the necessary information and telephone numbers for further questions.

5.7 Data collected and data analyses

5.7.1 Behavioural and self-report data

In addition to the persistent and repeatedly self-reported masses, immunological parameters and the behavior during the standardized positive interaction at laboratory visit 1 are recorded as described above.

The behavioral and self-report data are evaluated at the Department of Clinical Psychology and Psychotherapy of the Psychological Institute of the University of Zurich (Head: Prof. Dr. U. Ehlert).

5.7.2 Biological data

The hormonal data (salivary cortisol) and the salivary enzyme alpha amylase will be analyzed in the psychobiological laboratory of the Department of Clinical Psychology and Psychotherapy of the Psychological Institute of the University of Zurich (Head: Prof. Dr. U. Ehlert).

The following parameters will analyzed as wound healing parameters:

- Size of the wound (photographed with measuring tape immediately after wound application and 7 days after wound application)
- Trans-epidermal Water Loss (TEWL; using an evaporimeter before wound application, immediately after removal of the epidermis, 24 hours after removal of the epidermis, 7 days after removal of the epidermis)
- Epithelialisation (by microscopy before wound application, immediately after removal of the epidermis, 24 hours after removal of the epidermis, 7 days after removal of the epidermis)
- Mediators of wound healing: cytokines in the wound fluid (TNF alpha, IL1, IL6; analysis of the wound fluid 1) from two blisters immediately after application of the suction blisters, 2) from two intact blisters 24 hours after application of the suction blisters).

The evaluation of the data on wound size, TEWL and epithelialization will be carried out in the Dermatology Department of the USZ. The cytokine concentrations are analyzed by the Cytolab laboratory (Mr Adrian Urwyler, CH-8108 Dällikon). All data will be analyzed in strict compliance with the anonymization of the subjects (see below).

5.7.3 Statistical data analyses

The primary endpoint of sub-study 1 is reached when 100 couples have been examined.

The group differences analyzed in sub study 1 (oxytocin vs. placebo, men vs. women) in relation to the main outcome variable wound healing will be evaluated using analyses of variance (ANOVAs). Associations between the repeated measures data of the EMA part of the study and the main outcome variable wound healing will be analyzed using hierarchical linear models (HLM) to account for the dependent data structure between partners.

The significance level of the data analysis is set at 5%. Individual missing data points are replaced by the group-specific mean value. The data of prematurely discontinuing study participants are analyzed for statistically significant differences with the remaining group.

All psychobiological data will only be analyzed after completion and termination of the sub-study in order to minimize inter-assay variance. Self-report and behavioral data will be analyzed in 6-month tranches.

6 Study design and study procedure, sub-study 2

Title: Eye-tracking of oxytocin and couple-specific behaviour

6.1 Research question

Sub-study 2 investigates whether oxytocin is specifically involved in the perception of couple- and/or attachment-specific stimuli in humans, compared to stimuli without a social context. Animal studies show a bonding and even "fidelity"-promoting aspect of oxytocin (Young & Wang, 2001; Insel & Young, 2001). In humans, oxytocin could be involved in close non-sexual attachment relationships (e.g. close friendships, parent-child bonds, bonds between relatives) as well as in sexual relationships, but this has not yet been investigated differentially. Animal studies also suggest a gender-specific effect of oxytocin (with stronger effects in female animals). A gender effect of oxytocin on the perception of and interest in bonding or sexual stimuli has not yet been investigated in humans.

6.2 Hypotheses

We hypothesise that oxytocin, compared to placebo, increases interest in attachment-specific images and sexual images compared to non-social neutral control images. Furthermore, we would like to investigate a possible gender difference in relation to this effect. Since almost all studies on the effect of oxytocin have been conducted on men, we cannot yet make any assumptions about the direction of the effect in our study and formulate the hypothesis of the gender effect unspecifically.

6.3 Main target parameters

The main target parameter in sub-study 2 is the interest in attachment-specific images or sexual images compared to non-social neutral control images. This interest is measured by the time spent looking at the images and by focusing on central image regions using eye tracking. The self-reported evaluation of the images is recorded as a secondary target parameter.

6.4 Study design

Sub-study 2 will be conducted in accordance with ICH-GCP and in a randomized double-blind and placebo-controlled design.

6.4.1 Study sample and power analyses

In sub-study 2, 88 people (44 women, 44 men) will be examined who will receive either oxytocin nasal spray or placebo nasal spray. The probability of allocation to one of the two groups is 50%. The participating women will be analysed twice during two different cycle phases. Power analyses suggest a sample size of N=82 for sub-study 2 with regard to the main outcome variable preference (gaze duration) (effect d =.60; power = .85, alpha = .05). With an assumed effect f = .50, alpha = .05 and an N = 20, a power of ß = .85 can be assumed for the repeated measurement of the female participants. The total N is therefore set to N=88 in order to compensate for any dropouts.

6.4.2 Inclusion and exclusion criteria

The subjects must fulfil the following *inclusion criteria:*

- Women and men
- Between 25 and 45 years
- Willingness to attend one or two laboratory appointments
- Be healthy, e.g. have no acute, chronic physical, neurological, psychiatric or somatic illnesses
- Be a non-smoker (up to 5 cigarettes per day)
- fluent German
- Women: have a regular menstrual cycle (between 24 - 35 days)

Exclusion criteria:

- Previous participation in other studies on the topic of couple interactions or participation in another clinical study within the last 4 weeks before inclusion
- Accident with neurological sequelae
- Allergies or a history of known hypersensitivity to one of the drugs used or their ingredients, or to drugs with a similar chemical structure
- Taking medication
- Drug use, addiction or other illnesses that do not allow the person concerned to the nature, scope and possible consequences of the clinical trial
- Overweight or underweight

- Daily alcohol consumption (> 60g alcohol, equivalent to approx. 7.5 dl wine or 2 litres beer per day)
- No participation of the Investigator or his/her family members, employees or other dependent persons
- Women: hormonal contraception
- Women: Pregnancy (for instructions on the double barrier to prevent pregnancy, see subject information) or intention to become pregnant during the course of the study
- Women: Breastfeeding

These criteria are queried with the interested participants in a telephone interview. No personal details are recorded during this telephone interview, so that no personal data can be linked to the inclusion or exclusion criteria before the consent form is signed. An appointment for the study is arranged with interested and suitable participants, at which the inclusion and exclusion criteria are discussed again and the participants sign the declaration of consent.

For this study, it is essential that certain things are observed and adhered to, as these activities or factors influence the measured values and render them unusable. For this reason, the test subjects were asked to do so immediately before the study:

- Do not drink coffee or other caffeinated drinks (cola, energy drinks etc.), black tea or alcohol in the afternoon of the laboratory appointment (from approx. 3.00 pm),
- Do not engage in excessive sport on the day of the examination,
- Do not smoke immediately before the laboratory appointment,
- No physical exertion (cycling etc.) immediately before the laboratory appointment,
- Do not eat anything heavy immediately before the lab appointment.

The test subjects are recruited via circular mails from the mailing list for the recruitment of test subjects of the Psychological Institute of the University of Zurich, the description of the study in public media (ronorp, 20Minuten, NZZ), notices and websites for the recruitment of test subjects and receive CHF 50.00 per visit as compensation for participation.

6.4.3 Procedure of the experiment

After a telephone interview in which inclusion and exclusion criteria are clarified, the first laboratory appointment is arranged with interested test subjects. After reading the test person information and signing the written declaration of consent at the first appointment, the test

persons receive a test person code and are given an individualised link to the study website. This link will take the test subjects to questionnaires on personality, relationship quality and chronic stress, which they can complete online. The study itself will take place in the study rooms of the Psychological Institute of the University of Zurich (Binzmühlestr. 14, 8050 Zurich Örlikon).

Study information and declaration of consent:

The potential study participants are informed about the procedure and aim of the study by means of the written information for volunteers. It is also explained to them that a pregnancy excludes participation.

Admission procedure:

Study participants will be asked to give their voluntary consent in writing. The enrolment examination is carried out according to the following criteria:

- Assignment of a subject number (in ascending order)
- Review of the inclusion and exclusion criteria
- Multi-drug test (urine test)
- For women: last menstruation + pregnancy test (standard urine test)

After fulfilment of the inclusion criteria, the investigator gives approval to start the study.

Oxytocin/placebo administration:

The subjects are then asked to take the nasal spray (24 IU = 3 sprays of 4 IU in each nostril, oxytocin or placebo in a double-blind design).

Eye-tracking:

45 minutes later, the test subjects will be shown a total of 90 images depicting attachment motives, explicit sexual acts or control motives in a neighbouring room after an exercise run on the computer. The test subjects can look at these pictures for as long as they like and then move on to the next picture. After each picture, they rate the attractiveness of the respective picture using a visual analogue scale (VAS). While they are looking at the images, their eye movements are recorded with an eye-tracking device using infrared. This procedure is non-

invasive, painless and completely harmless for the participants. The fixation points and fixation durations of the eye movements are recorded for each image. The length of time each image is viewed is also recorded. The images are presented using the "presentation" programme. The participants are also asked to rate each picture. After they have viewed and rated the pictures, the debriefing takes place, the subjects receive their compensation and the experiment is completed. The entire experiment takes about 2.5 hours.

6.5 Research methods

6.5.1 Psychological examination methods

Self-assessed health (General Health Questionnaire, GHQ-12, Banks, 1983), personal information (standardised anamnesis questionnaire on housing and living situation, socio-economic status, etc.), personality traits (NEO-FFI, Borkenau & Ostendorf, 2008), sexual dysfunctions or experiences with - and possibly aversions to - pornographic images (German translations of the Brief Index of Sexual Function, BISF, Taylor et al, 1994, and for women the Female Sexual Function Index, FSFI, Rosen et al., 2000), as well as relationship satisfaction (German version of the Relationship Assessment Scale, RAS, Hassebrauck, 1991) are completed after signing the consent ständniserklärung. The assessment of the pictures is asked after each picture using a visual analogue scale.

6.5.2 Physiological examination methods: Oxytocin application

Oxytocin or placebo is taken intranasally (24 IU = 3 sprays of 4 IU in each nostril) by the subjects after signing the informed consent form and completing the questionnaires in a double-blind design.

Compliance

The compliance of the test subjects with regard to taking their medication can be observed directly in sub-study 2.

Pre-treatment and concomitant therapy

No other treatments or medications are permitted during the study. Any concomitant therapies will be requested at the start of the study. All possible concomitant and/or emergency medication(s) must be documented in the CRF.

Packaging, labelling and delivery

The test preparation is given to the test subjects in the form of clearly labelled nasal sprays. The labels on the nasal sprays provide instructions on the dosage in which the nasal spray should be taken (see labels in the appendix). The study medication will be provided by KAZ. The nasal sprays will be collected in tranches of 10 sprays each by the study coordinator and stored in a lockable refrigerator in a restricted-access room at the Institute of Psychology until the experiment. Upon receipt of the study medication/study material, an inventory is taken, a Drug Receipt Log is filled out and signed by the person who received the material. A thorough check is made to ensure that the consignment contains all the items on the delivery note. If the delivery contains defective or unusable study medication, this is recorded in the study documents. Regular drug reconciliation regarding allocation, administration and remaining study medication will be performed, documented with the Drug Reconciliation Form, and dated and signed by the study team. At the end of their participation in the study, the nasal sprays will be collected and documented by the study management. The returned nasal sprays will be kept until data collection is completed to enable any cross-checks with regard to blinding.

Blinding

The study is a placebo-controlled double-blind study. This means that the placebo contains all the same ingredients as the original medication - except for the active ingredient. The preparation of the placebo and blinding of the study medication is carried out by KAZ. The preparation of the placebo and blinding of the study medication is carried out by KAZ (GMP certificate see appendix). An assistant from the Chair of Clinical Psychology and Psychotherapy (lic. phil. Jana Campbell) who is not involved in the study will carry out the randomisation using the "Research Randomizer" program and submit the randomisation table to KAZ. The KAZ will hand over the table sealed in an envelope to the study management after the preparation of the test sample. This allows the investigator to unblind the code in an emergency. The code should only be broken in an emergency if the identity of the preparation must be known to the investigator in order to ensure the medical care of the participating person. After completion of data collection, the blinding is cancelled.

Storage conditions

The study medication is stored refrigerated in the KAZ. The unopened medication has a shelf life of three years. As the medication is not opened for blinding, all medication can be ordered and stored in one batch.

Return or destruction of the study medication

At the end of the study, a final review of the transport, dispensing and whereabouts of the study medication is carried out. This is documented with the Drug Reconciliation Form, dated and signed. Any discrepancies will be investigated, clarified and documented before any remaining study medication is destroyed. The destruction of the study medication is then recorded in the study documents.

6.6 Collected data and data analyses

6.6.1 Behavioural and self-report data

In addition to the persistent and self-reported masses , eye-tracking data is collected as behavioural data, as described above. The data is analysed using the program supplied with the eye tracker ("Tobii Studio™ Analysis Software"). For this purpose, different areas of interest (AOI) of the images are compared with each other in terms of gaze duration and frequency. The self-report and eye-tracking data are analysed at the Department of Clinical Psychology and Psychotherapy of the Institute of Psychology at the University of Zurich (Head: Prof. Dr. U. Ehlert). The evaluation of this data is carried out in strict compliance with the anonymisation of the test subjects (see below).

6.6.2 Statistical data analyses

The primary endpoint of sub-study 2 is reached when 88 subjects have been examined.

The group differences analysed in sub-study 2 (oxytocin vs. placebo, men vs. women, in women: Follicular phase vs. luteal phase) are analysed using (repeated measures) analyses of variance (ANOVAs).

The significance level of the data analyses is set at 5%. Individual missing data points are replaced by the group-specific mean value. The data of prematurely discontinuing study participants are analysed for statistically significant differences with the remaining group.

Self-report and behavioural data are analysed in 6-month tranches.

7 Risk-benefits ratio and ethical considerations

Couple relationships and marriages are clearly associated with health and longevity and significantly better survival rates after health threats such as cancer and myocardial infarction. The psychobiological mechanisms underlying this protective effect of close relationships are not yet known. However, a buffering effect of closeness and social support during stress is suspected. A recently published study investigating the effect of social support and couple conflict behaviour on wound healing was also able to show that couple conflict and, in particular, hostile interaction during couple interactions delayed wound healing. It remains to be found out which neuronal mechanisms cause this effect in the central nervous system.

Previous studies have shown that the nonapeptide hormone oxytocin is particularly involved in the regulation of bonding behaviour and pair interaction at the central nervous level and attenuates the neuroendocrine stress response. Data from non-human mammals suggest that oxytocin-induced suppression of the HHN axis supports wound healing. This effect has not yet been studied in humans, but it is hypothesised that the same interaction mechanisms of stress, oxytocin and wound healing are present as in non-human mammals.

Wound healing in the context of stress vs. social support has very important clinical implications, for example for recovery after surgery, but also for wounds in everyday life. Therefore, the identification of mechanisms that may influence the relationship between stress, social interaction and wound healing on a neuronal level is highly relevant for behavioural medicine disorders and health in general.

7.1 Ethical aspects

The project and its sub-studies will be conducted in accordance with the ethical guidelines of the Helsinki Declaration and comply with the ICH-GCP standard. A drug will be used that is authorised on the Swiss market and for which no serious side effects have been reported in previous studies. The following measures will be taken to ensure subject safety, confidentiality and data integrity:

7.2 Confidentiality of respondent data

All subjects are assigned an identification number (in ascending order) when they first register. All materials collected will be labelled with this number. Only the consent form will contain both the name and the number and will be stored separately from all data in a sealed box.

No data will be analysed by name, only by subject number, and there will be no way to link the name to the ID number other than through the consent form. All psychological and biochemical data will only be identifiable by code number. No publication of this project will disclose the names of the subjects.

7.3 Potential risks and protection against risks

The risks to subjects in this project and the sub-studies are low. Nevertheless, there are areas of potential risk associated with intranasal oxytocin administration and bladder wound induction.

7.3.1 Syntocinon (oxytocin) nasal spray

Syntocinon-Spray® is a well-tolerated medicine that is widely used in obstetrics. The nasal spray may cause slight irritation of the nasal mucosa with increased secretion. In rare cases, allergic reactions, nausea, vomiting or headaches have been reported, as well as abdominal pain due to uterine contractions. Occasionally, oxytocin causes an increase in blood pressure, rarely a drop in blood pressure, which may be accompanied by reddening of the skin and reflex tachycardia. However, studies on healthy volunteers have so far shown no side effects following the administration of oxytocin nasal spray. We do not expect any side effects when using the nasal spray. We ask the test subjects to report any symptoms to us immediately so that they can be treated.

7.3.2 Suction blister application, measurement of wound fluid and measurement of the wound

The application of the suction blisters to the arm results in a small wound. This wound may cause slight pain. Removing the wound fluid can also be painful. In very rare cases, the wound may become inflamed, be painful for longer and leave scars. No medical side effects are to be expected when the wound is measured after one week.

We will apply the suction blisters and measure the wound fluid in accordance with international standards and under sterile conditions. In addition, the test subjects are asked to report any symptoms or pain to us immediately so that they can be treated.

7.3.3 For women of childbearing age

As Syntocinon spray can lead to uterine contractions towards the end of a pregnancy and can trigger milk production, it must not be used in pregnancy except for these medical implications. We therefore carry out a standard pregnancy test on all female participants before the start of the study. In the study, a double-barrier method of contraception is used to prevent pregnancy, i.e. the study participants use two reliable methods of contraception during the study (in sub-study 1: pill in combination with -, otherwise double mechanical contraception, e.g. diaphragm, IUD etc. in combination with condoms). Patients who become pregnant during the study must inform their doctor immediately and may not continue to participate in the study. In this case, they are asked to consult a specialist doctor so that specialist medical care is ensured and to provide information on the course and outcome of the pregnancy (see also 7.5.1). Women who are breastfeeding are excluded from participation in the study. In order to rule out the possibility of pregnancy occurring during the study in everyday life, we examine all our participants during the luteal phase (day 16 - 28 of the menstrual cycle).

7.3.4 For men

There are no standard studies on the effect of oxytocin on sperm and reproductive function. It is therefore important to use a double barrier to prevent pregnancy during the study and up to three months after participation in the study (in sub-study 1: pill in combination with -, otherwise mechanical contraceptive methods, e.g. diaphragm, IUD etc. in combination with condoms, see above), because damage to the sperm cannot be ruled out.

7.3.5 Measurements in everyday life

The psychological measurements in everyday life (between laboratory appointment 1 and the wound measurement on appointment 3), in which we use a handheld computer to assess mood and current social interactions, do not harbour any medical risk.

The saliva measurements in everyday life (between laboratory appointment 1 and the wound measurement on appointment 3), in which we ask the test subjects to collect saliva samples 6 times a day using saliva vials, do not harbour any medical risk.

7.3.6 Stimulus material

In sub-study 2, the test subjects will see explicit images of couples having sex. These images do not contain any violence. If the subjects feel uncomfortable looking at these images, they can stop the study at any time without giving a reason and without negative consequences. Before the start of the study, we will ask the participants about their experiences and any aversions to sexual images and exclude them from the study if they do not wish to view the images.

7.3.7 Eye tracking

There are no known unpleasant side effects of eye tracking.

7.3.8 Psychological questionnaires

We will use questionnaires to ask the respondents about personality traits and also repeatedly about their mood. We are not aware of any unpleasant side effects of such questioning.

Subjects will be fully informed of all procedures and risks and will sign a consent form before participating in the study. The consent form will include the objectives and risks, as well as the fact that subjects have the right to withdraw from the study at any time. Participation in the study is voluntary and subjects may refuse or withdraw at any time. Under certain circumstances, subjects may also be excluded from the study without their consent if the sponsor (Dr Beate Ditzen) or the investigator (Dr Severin Läuchli) feel that it is not in the subjects' best interests to continue with the study or if the procedure is not followed. Subjects are instructed to inform the principal investigators immediately if any adverse events occur in connection with their participation in the study. In such cases, a physician will be on hand immediately to investigate and treat the symptoms and the management of AEs (see above) will take effect.

7.4 Efficacy and safety variables

The efficacy and safety of the investigational product is recorded in both sub-studies using the dependent variables described in each case. Any further effects are queried and documented daily by the test subjects using a checklist, in sub-study 1 via the contact using a handheld computer, in sub-study 2 directly during the contact in the laboratory.

7.5 (Serious) adverse events, side effects

7.5.1Adverse event (AE)

An adverse event (AE) is any untoward occurrence that happens to a person participating in the study who has been administered an investigational product and that is not necessarily causally related to this treatment. These can be illnesses, signs of illness, clinically significant laboratory values or symptoms that occur or worsen after the patient has been included in the study.

AEs observed by the investigator or reported by the participant must be recorded in the CRF for the entire duration of the study (from the signing of the informed consent form to the last protocol-specific procedure, regardless of the study medication).

Sufficient information will be obtained from all AEs to allow classification (e.g. as SAE) and to determine the causal relationship to the study medication or study treatment.

Whenever possible, concomitant diseases or conditions that necessitate a therapeutic or diagnostic procedure should be recorded as AEs. Surgery or other invasive procedures that were planned before the start of the study do not have to be recorded as AEs. Such planned procedures are recorded in the CRF by the investigator at the first visit (baseline visit/screening visit).

Pregnancy per se is not an AE. Adverse events that occur in connection with pregnancy must be documented like all other AEs. Pregnancy should be confirmed with an appropriate laboratory test. All pregnancies occurring during the study or up to 30 days after the last receipt of study medication must be reported to the sponsor-investigator within one working day (using a Pregnancy Initial Report Form). If the final visit takes place more than 30 days after the last receipt of study medication, all AEs occurring up to the final visit must be reported. The sponsor-investigator is further required to follow the pregnancy until birth and to document the health status of the newborn with a Pregnancy Follow-Up Report Form.

7.5.2 Adverse drug reaction (ADR)

An adverse reaction (AR) is any adverse and unintended reaction to an investigational medicinal product, regardless of its dosage. Classification as a reaction is made if a connection between the event and the investigational medicinal product is at least considered possible.

An Unexpected Adverse Reaction (UAR) is an adverse reaction that is not consistent in type or severity with the available information on the investigational medicinal product.

7.5.3 Serious Adverse Event (SAE)

An SAE or Serious Adverse Reaction (SAR) is any adverse event or side effect that

- leads to death or
- is life-threatening or
- leads to permanent or severe disability or invalidity, or
- requires inpatient treatment or its extension, or
- leads to congenital malformations or birth defects or
- is medically significant for other reasons (event requiring medical intervention to prevent an outcome considered serious).

7.5.4 Suspected Serious Unexpected Adverse Reaction (SUSAR)

A suspected case of an Unexpected Serious Adverse Reaction is referred to as a Suspected Unexpected Serious Adverse Reaction (SUSAR). A serious adverse reaction is unexpected if it is not listed in the Information for healthcare professionals/ IB.

7.6 Documentation of (S)AEs

The investigator will ask the participant at each visit and via handheld computer how they are feeling. It is the responsibility of the investigator to document all adverse events, including intercurrent illnesses, in the CRF. The time from which AEs are documented is the signature of the informed consent form.

If an adverse event occurs, the affected person must be observed in any case, regardless of the causal relationship between the event and the investigational product, until the symptoms have subsided or pathological laboratory values have returned to the initial values, or until, in the opinion of the principal investigator, no further findings are to be expected. If the adverse event results in a persistent sequela, this must be classified as an SAE and documented accordingly at the end of the study. All findings and results must be documented on the corresponding page for Adverse Events in the CRF.

The following information is required:

- Type of adverse event (sign, symptom or disease, diagnosis if possible),
- Assessment of (worsening of existing) concomitant diseases as an adverse event,
- Differentiation (serious/not serious)
- Start and end of occurrence,
- Intensity (mild = easily tolerated, moderate = affects daily activities, severe = daily activities/work not possible; CTC criteria only recommended in oncological studies),
- Causality to the investigational medicinal product,
- Measures relating to the investigational medicinal product or actions to restore or improve the well-being of the person concerned,
- Outcome of the event.

The following points must be strictly adhered to when documenting the SAEs:

- the SAE must be documented on the corresponding AE page in the CRF and on the SAE form,
- Each SAE must be reported as completely as possible. (in the event of death, an autopsy should be performed if possible and the report made available to the Principal Investigator),
- the person responsible for monitoring must check the data at the test centre for completeness and ensure that the information in the SAE report matches the information in the database and other data sources.

Cases of overdose, misuse, application errors, etc. should be documented, even without an AE occurring.

7.7 Assessment of (Serious) Adverse Events

The Investigator will review documented AEs or abnormal test results as soon as possible to determine if

- the abnormal test result is to be classified as an AE
- there is a possibility that the study medication or the study treatment triggered the AE, and
- whether the criteria for an SAE are met.

The causal relationship of the (S)AE with the investigational product is determined by the investigator according to the following definitions:

Unlikely relation: the temporal relationship with the administration of the study medication makes a causal relationship unlikely and other drugs, chemicals or underlying diseases offer a plausible explanation.

Possible relation: the temporal relationship between administration of the study medication and occurrence of the (S)AE is given, but other drugs, chemicals or underlying diseases offer a plausible explanation, or information from discontinuation of the study medication is missing or unclear

Likely relation: the temporal relationship between the administration of the study medication and the occurrence of the (S)AE is given, and other drugs, chemicals or underlying diseases do not provide a credible explanation, and the discontinuation of the study medication (dechallenge) is followed by an appropriate response. Information about a rechallenge is not necessary to fulfil this definition.

Certain relation: the temporal relationship between the administration of the study medication and the occurrence of the (S)AE is given, and other drugs, chemicals or underlying diseases offer no plausible explanation, and discontinuation of the study medication (dechallenge) is followed by a clinically plausible reaction. The event is pharmacologically **and** phenomenologically definite, demonstrated by rechallenge if necessary.

7.8 Reporting of serious adverse events (SAEs)

The sponsor-investigator is responsible for reporting SAEs to Swissmedic and ethics committees in accordance with the following details:

- compliance with the regulatory requirements of Swissmedic for the reporting of unexpected SAEs for which the causal relationship with the study medication cannot be excluded.
- reporting fatal or life-threatening SAEs to the authorities (Swissmedic/FOPH) if they are suspicious, unexpected and related to the study medication (SUSAR)

  - without delay but no later than **7 calendar days** after realisation that the event is to be considered a SUSAR.

- Follow-up information on the SUSAR within a further **8 calendar days**.

- reporting non-fatal or non-life-threatening SAEs to the authorities (Swissmedic/FOPH) if they are suspicious, unexpected and related to the study medication (SUSAR)

  - without delay, but no later than **15 calendar days** after realisation, that the event is to be considered a SUSAR.

- the sending of annual safety reports, for the first time one year after notification to Swissmedic. These reports should contain

  - A brief, critical summary of the safety profile of the study medication and safety issues that have arisen;

  - A list of all SUSARs that have appeared in Switzerland and internationally;

  - If possible, all adverse reactions (ARs) that have occurred internationally.

- reporting fatal SAEs to the Ethics Committee:

  - immediately, i.e. **within 24 hours**.

- reporting fatal and life-threatening SAEs to the ethics committee if they are suspicious, unexpected and related to the study medication (SUSAR):

  - without delay but no later than **7 calendar days** after realisation that the event is to be considered a SUSAR.

  - Follow-up information on the SUSAR within a further **8 calendar days.**

- The reporting of non-fatal or non-life-threatening SAEs to the Ethics Committee if they are suspicious, unexpected and related to the study medication (SUSAR)

  - without delay, but no later than **15 calendar days** after realisation, that the event is to be considered a SUSAR.

An unexpected SAE corresponds to an AE whose nature or severity is not consistent with the product information (, IB).

7.9 Follow-up of (serious) adverse events

The health status of participating persons who discontinue the study (regularly or prematurely) due to

- reported, persistent SAE, or
- persistent adverse event, e.g. laboratory values or alarming vital signs,

is examined at a follow-up visit. This visit takes place up to 30 days after the end of the study treatment. The follow-up information is recorded on the corresponding AE page of the CRF. The corresponding medical file must be available for inspection upon request.

Efforts to find lost to follow-up patients must be made and documented. In the case of minor AEs, telephone calls may be sufficient.

New SAEs or pregnancies must be reported using the appropriate forms and entered in the CRF within 30 days of the last dose of study medication. The reporting obligation is extended until the final visit if this takes place later than 30 days after the last treatment with the study medication.

Follow-up visits may also be necessary at the discretion of the investigator, even if the participant does not have an AE at the end of the study. The information of such visits only needs to be documented in the medical record, not in the CRF.

8 Data Quality Assurance

Monitoring and quality assurance audits are carried out as part of the clinical trial.

8.1 Monitoring

Regular monitoring during the study checks the collection of data, ensures compliance with the protocol, ensures the accuracy of the data and enables errors to be detected at an early stage. The sponsor is responsible for organising professional, independent monitoring.

The documents relating to the study, CRFs, study-relevant original patient files, laboratory and medical test results must be available for inspection by the monitor. The monitor reviews CRFs and patient consent forms. The accuracy of the data is verified by reviewing the above documents.

The Centre for Clinical Research (CTC) of the University Hospital Zurich will ensure monitoring at the trial site. The monitoring will be performed according to ZKF/CTC SOPs. The type and frequency of monitoring will be defined in the separate monitoring plan.

8.2 Audits and inspections

Audits or inspections may be carried out by the authorities (Swissmedic, EC) to ensure that the study is conducted in accordance with GCP guidelines. The auditor/inspector is given access to all medical records, study-relevant documents and correspondence, as well as patient consent forms.

The Investigator ensures that the persons responsible for the audit/inspection have access to medical records and that any questions that arise are answered. All persons involved treat patient data as strictly confidential.

8.3 Specification of source documents

The following documents are considered source documents:

- SAE worksheets
- Notes from nursing staff, study coordinators
- Medical records from other institutes or other hospitals, or findings, doctor's letters or correspondence with other institutes/hospitals if the patient was treated there during the study or during the follow-up phase.

The source documents must be available at the study centre in order to prove the existence of study participants and the completeness of the data. Source documents must contain original documents relevant to the study as well as the participant's medical history.

The following information should be included as a minimum in the source documents:

- Demographic data (age, gender)
- Details on inclusion and exclusion criteria
- Dated and signed patient consent form
- Dates of the visits
- Details of the medical history of physical examinations
- The efficacy and safety data specified in the protocol

- AEs and concomitant medication
- Results of relevant studies
- Lab printouts
- Details on dispensing and returning study medication
- Reasons for early resignation
- Randomisation number

8.4 Documentation and storage of data

The study is conducted strictly in accordance with the protocol. If changes become necessary, these must be recorded in a protocol amendment. All protocol amendments must be signed by the Principal Investigator and other Investigators (except for organisational changes).

For each participating person, the investigator keeps a data collection form (CRF) in which all study-relevant data of the participating person are entered. All data collected in this study must be entered in the CRF by appropriately authorised persons. All persons (participating persons, persons not suitable for the study, persons suitable for the study but not included) are documented in a screening log. In the proposed project, the data collection form will be set up in collaboration with the CTU using the "SecuTrial" system and managed electronically (eCRF, electronic Case Report Form, see contract between the data management of the CTU and Dr B. Ditzen in the appendix).

The investigator notes the participation on a special enrolment log (patient identification list). It is used to identify the participating persons at a later date and contains the patient number, full name, date of birth and date of enrolment in the clinical trial. The patient identification list remains at the trial centre after completion of the trial.

Furthermore, it must be ensured that the person responsible for the documentation in the CRF can be identified.

It is the responsibility of the investigator to ensure that all data collected as part of the study are entered correctly and completely into the database created specifically for this study.

Corrections in the eCRF may only be made by authorised persons or by the responsible investigator and must be justified. Corrections are recorded in such a way that the old entry can still

be retrieved. All data and corrections are automatically logged with the date, time and the person making the entry.

All essential documents of the clinical trial must be retained by the sponsor for at least 10 years after the end or discontinuation of the clinical trial. The sponsor must archive the study-relevant records and documents in accordance with the statutory provisions.

Records and documents related to the study or the allocation of investigational medicinal products (e.g. data collection forms, consent forms, medication distribution lists and other relevant documents) must be kept by the investigators for at least 10 years (VKlin Ar. 25).

8.5 Data protection and confidentiality

The collection, disclosure, storage and evaluation of personal data within this clinical study is carried out in accordance with the Swiss legal provisions of the Federal Act on Data Protection (FADP) and the Ordinance on the Disclosure of Professional Secrets in the Field of Medical Research (ODA). The prerequisite for this is the voluntary consent of the participating persons within the framework of the declaration of consent prior to participation in the clinical study.

Medical information collected from participants during this study is strictly confidential and may not be disclosed to third parties. Confidentiality is further ensured by the use of participant identification code numbers, which are assigned to the treatment data.

If the participant has given their consent, such medical information may be passed on to the family doctor or other attending physicians to ensure the patient's well-being.

Data collected as part of this clinical trial are available for review or inspection by monitors, the relevant ethics committee or the competent authority.

9 Insurance

The insurance is covered by the liability insurance for the University of Zurich, which was taken out specifically for this study. This covers all damages in connection with the study. Study participants must strictly adhere to the instructions of the study personnel. Furthermore, they may not undergo any other medical treatment during the clinical trial without the consent of the investigator (with the exception of emergencies). They must inform the investigator

immediately of any emergency treatment. If health problems or other damage occur during or after participation in the study, the investigator must be informed. A copy of the insurance certificate will be filed in the trial centre folder.

10 Study registration

This study is registered in the local study register of the University Hospital Zurich ("Study Register USZ") and in the international register ClinicalTrials.gov (clinicaltrials.gov).

11 Publications

The investigator will make every effort to publish the data of this study in a medical journal after the statistical analysis.

12 Timetable

- From March 2011 Recruitment of test subjects
- From May 2011 Experimental phase of sub-study 1
- From May 2011 Experimental phase of sub-study 2
- From March 2013 Completion of the study, evaluation of the results

13 signatures

The following persons agree to the content of the clinical study and confirm this with their signature. Changes that affect the responsibility of each of the signatories must be reported immediately.

Sponsor-Investigator

I hereby confirm that I have read and understood this protocol and accept it in its entirety. I undertake to ensure that the persons brought into the study by my centre are treated, observed and documented in accordance with the provisions of this protocol.

Place, date	Signature, Dr Beate Ditzen

Investigator

I hereby confirm that I have read and understood this protocol and accept it in its entirety. I undertake to ensure that the subjects examined in this study are examined and treated in accordance with the study protocol.

Place, date	Signature, Dr Severin Läuchli

This protocol was drawn up in accordance with the criteria of the Helsinki Declaration, ICH-GCP and the corresponding SAMS guidelines.

14 References

Amico, J. A., Mantella, R. C., Vollmer, R. R., & Li, X. (2004). Anxiety and Stress Responses in Female Oxytocin Deficient Mice. *J Neuroendocrinol, 16*(4), 319-324.

Baumgartner, T., Heinrichs, M., Vonlanthen, A., Fischbacher, U., & Fehr, E. (2008). Oxytocin Shapes the Neural Circuitry of Trust and Trust Adaptation in Humans. *Neuron, 58*(4), 639-650.

Berkman, L. F., & Syme, S. L. (1979). Social networks, host resistance and mortality: A nine-year follow-up study of Alameda County residents. *American Journal of Epidemiology, 109*, 186-204.

Bodenmann, G. (2008). *Dyadic Coping Inventory (DCI). Test manual.* Berne, Göttingen: Huber & Hogrefe.

Borkenau, P. & Ostendorf, F. (2008). *NEO Five-Factor Inventory according to Costa and McCrae (NEO-FFI). Manual (2nd, newly standardised and completely revised edition).* Göttingen: Hogrefe.

Born, J., Lange, T., Kern, W., McGregor, G. P., Bickel, U., & Fehm, H. L. (2002). Sniffing neuropeptides: a transnasal approach to the human brain. *Nature Neuroscience, 5*(6), 514-516.

Buchner, A., Faul, F., & Erdfelder, E. (1998). A priori, post-hoc, and compromise power analyses for MS-DOS (German Version). from http://www.psycho.uni-duesseldorf.de/aap/projects/gpower/index.html

Burman, B., & Margolin, G. (1992). Analysis of the association between marital relationships and health problems: An interactional perspective. *Psychol Bull, 112*(1), 39-63.

Carter, C. S. (1998). Neuroendocrine perspectives on social attachment and love. *Psychoneuroendocrinology, 23*(8), 779-818.

Carter, C. S., DeVries, A. C., & Getz, L. L. (1995). Physiological substrates of mammalian monogamy: the prairie vole model. *Neurosci Biobehav Rev, 19*(2), 303-314.

Chandra, V., Szklo, M., Goldberg, R., & Tonascia, J. (1983). The impact of marital status on survival after an acute myocardial infarction: a population-based study. *Am J Epidemiol, 117*(3), 320-325.

Christian, L. M., Graham, J. E., Padgett, D. A., Glaser, R., & Kiecolt-Glaser, J. K. (2006). Stress and wound healing. *Neuroimmunomodulation, 13*(5-6), 337-346.

Clark, R. A. F. (1996). *The molecular and cellular biology of wound repair*. New York: Plenum Press.

Coan, J. A., Schaefer, H. S., & Davidson, R. J. (2006). Lending a hand: social regulation of the neural response to threat. *Psychol Sci,17*(12), 1032-1039.

Coyne, J. C., Rohrbaugh, M. J., Shoham, V., Sonnega, J. S., Nicklas, J. M., & Cranford, J. A. (2001). Prognostic importance of marital quality for survival of congestive heart failure. *Am J Cardiol, 88*(5), 526-529.

Dearman, R. J., Bhushan, M., Cumberbatch, M., Kimber, I., & Griffiths, C. E. (2004). Measurement of cytokine expression and Langerhans cell migration in human skin following suction blister formation. *Exp Dermatol, 13*(7), 452-460.

Detillion, C. E., Craft, T. K., Glasper, E. R., Prendergast, B. J., & DeVries, A. C. (2004). Social facilitation of wound healing. *Psychoneuroendocrinology, 29*(8), 1004-1011.

DeVries, A. C., Craft, T. K., Glasper, E. R., Neigh, G. N., & Alexander, J. K. (2007). 2006 Curt P. Richter award winner: Social influences on stress responses and health. *Psychoneuroendocrinology, 32*(6), 587-603.

Ditzen, B., & Heinrichs, M. (2007). Psychobiological mechanisms of social support. *Journal of Health Psychology, 15*(4), 143-157.

Ditzen, B., Hoppmann, C., & Klumb, P. (2008). Positive Couple Interactions and Daily Cortisol: On the Stress-Protecting Role of Intimacy. *Psychosom Med.*

Ditzen, B., Neumann, I. D., Bodenmann, G., von Dawans, B., Turner, R. A., Ehlert, U., et al. (2007). Effects of different kinds of couple interaction on cortisol and heart rate responses to stress in women. *Psychoneuroendocrinology, 32*(5), 565-574.

Ditzen, B., Schaer, M., Bodenmann, G., Gabriel, B., Ehlert, U., & Heinrichs, M. (2009). Intranasal Oxytocin Increases Positive Communication and Reduces Cortisol Levels during Couple Conflict. *Biol Psychiatry, accepted*.

Ditzen, B., Schmidt, S., Strauss, B., Nater, U. M., Ehlert, U., & Heinrichs, M. (2008). Adult attachment and social support interact to reduce psychological but not cortisol responses to stress. *Journal of Psychosomatic Research, 64*(5), 479-486.

Domes, G., Heinrichs, M., Glascher, J., Buchel, C., Braus, D. F., & Herpertz, S. C. (2007). Oxytocin attenuates amygdala responses to emotional faces regardless of valence. *Biol Psychiatry, 62*(10), 1187-1190.

Domes, G., Heinrichs, M., Michel, A., Berger, C., & Herpertz, S. C. (2007). Oxytocin Improves "Mind-Reading" in Humans. *Biol Psychiatry, 61*(6), 731-733.

Ehlert, U., Erni, K., Hebisch, G., & Nater, U. (2006). Salivary alpha-amylase levels after yohimbine challenge in healthy men. *J Clin Endocrinol Metab, 91*(12), 5130-5133.

Enk, C.D., Mahanty, S., Blauvelt, A. & Katz, S.I. (1996). UVB induces IL-12 transcription in human keratino-cytes in vivo and in vitro. *Photochem Photobiol, 63*, 854-859.

Ewart, C. K., Taylor, C. B., Kraemer, H. C., & Agras, W. S. (1991). High blood pressure and marital discord: not being nasty matters more than being nice. *Health Psychol, 10*(3), 155-163.

Fahrenberg, J., Myrtek, M., Pawlik, K., & Perrez, M. (2007). Ambulatory assessment-monitoring behaviour in daily life settings: A behavioural-scientific challenge for psychology. *European Journal of Psychological Assessment, 23*(4), 206-213.

Gaab, J., Kappeler, A. & Ehlert, U. (2003). Generation of a questionnaire on functional somatic syndromes. Manuscript, University of Zurich.

Glasper, E. R., & DeVries, A. C. (2005). Social structure influences effects of pair-housing on wound healing. *Brain Behav Immun, 19*(1), 61-68.

Gmelch, S., Bodenmann, G., Meuwly, N., Ledermann, T., Steffen-Sozinova, O., Striegl, K. (2008). Dyadic Coping Inventory (DCI): A questionnaire to assess partner coping with stress. *Journal of Family Research, 20*(2),185-203.

Goodwin, J. S., Hunt, W. C., Key, C. R., & Samet, J. R. (1987). The effect of marital status on the stage, treatment, and survival of cancer patients. *Journal of the American Medical Association, 258*, 3125-3130.

Gretener, S. B., Lauchli, S., Leu, A. J., Koppensteiner, R., & Franzeck, U. K. (2000). Effect of venous and lymphatic congestion on lymph capillary pressure of the skin in healthy volunteers and patients with lymph edema. *J Vasc Res, 37*(1), 61-67.

Grewen, K. M., Anderson, B. J., Girdler, S. S., & Light, K. C. (2003). Warm partner contact is related to lower cardiovascular reactivity. *Behav Med, 29*(3), 123-130.

Gruenewald, T. L., Seeman, T. E., Ryff, C. D., Karlamangla, A. S., & Singer, B. H. (2006). Combinations of biomarkers predictive of later life mortality. *Proceedings of the National Academy of Sciences of the United States of America, 103*(38), 14158-14163.

Guastella, A. J., Mitchell, P. B., & Dadds, M. R. (2008). Oxytocin increases gaze to the eye region of human faces. *Biol Psychiatry, 63*(1), 3-5.

Guastella, A. J., Mitchell, P. B., & Mathews, F. (2008). Oxytocin Enhances the Encoding of Positive Social Memories in Humans. *Biol Psychiatry*, e-pub ahead of print.

Hahlweg, K. (1996). *Fragebogen zur Partnerschaftsdiagnostik (FPD) [Marriage diagnostic questionnaire]*. Göttingen: Hogrefe.

Heinrichs, M., Baumgartner, T., Kirschbaum, C., & Ehlert, U. (2003). Social support and oxytocin interact to suppress cortisol and subjective responses to psychosocial stress. *Biol Psychiatry, 54*(12), 1389-1398.

Hassebrauck, M. (1991). ZIP: An instrument for the assessment of satisfaction in couple relationships. *Journal of Social Psychology, 22*, 256-259.

Heinrichs, M., & Domes, G. (2008). Neuropeptides and social behaviour: Effects of oxytocin and vasopressin in humans. *Progress in Brain Research, in press*.

Heinrichs, M., Meinlschmidt, G., Neumann, I. D., Wagner, S., Kirschbaum, C., Ehlert, U., et al. (2001). Effects of suckling on hypothalamic-pituitary-adrenal axis responses to psychosocial stress in postpartum lactating women. *Journal of Clinical Endocrinology and Metabolism, 86*(10), 4798-4804.

Heinrichs, M., Meinlschmidt, G., Wippich, W., Ehlert, U., & Hellhammer, D. H. (2004). Selective amnesic effects of oxytocin on human memory. *Physiol Behav, 83*(1), 31-38.

Hendrick, S. S. (1988). A generic measure of relationship satisfaction. *Journal of Marriage and the Family, 50*, 93-98

Holst, S., Uvnas-Moberg, K., & Petersson, M. (2002). Postnatal oxytocin treatment and postnatal stroking of rats reduce blood pressure in adulthood. *Auton Neurosci, 99*(2), 85-90.

House, J. S., Landis, K. R., & Umberson, D. (1988). Social relationships and health. *Science, 241*(4865), 540-545.

House, J. S., Robbins, C., & Metzner, H. L. (1982). The association of social relationships and activities with mortality: prospective evidence from the Tecumseh Community Health Study. *Am J Epidemiol, 116*(1), 123-140.

Hubner, G., Brauchle, M., Smola, H., Madlener, M., Fassler, R., & Werner, S. (1996). Differential regulation of pro-inflammatory cytokines during wound healing in normal and glucocorticoid-treated mice. *Cytokine, 8*(7), 548-556.

Insel, T. R., & Hulihan, T. J. (1995). A gender-specific mechanism for pair bonding: oxytocin and partner preference formation in monogamous voles. *Behav Neurosci, 109*(4), 782-789.

Insel, T.R. & Young, L.J. (2001). The neurobiology of attachment. *Nat Rev Neurosci,2,* 129-136.

Jacinto, A., Martinez-Arias, A., & Martin, P. (2001). Mechanisms of epithelial fusion and repair. *Nat Cell Biol, 3*(5), E117-123. ^

Jacobi, U., Chen, M., Frankowski, G., Sinkgraven, R., Hund, M., Rzany, B., et al. (2004). In vivo determination of skin surface topography using an optical 3D device. Skin Res Technol, 10(4), 207-214.

Jutapakdeegul, N., Casalotti, S. O., Govitrapong, P., & Kotchabhakdi, N. (2003). Postnatal Touch Stimulation Acutely Alters Corticosterone Levels and Glucocorticoid Receptor Gene Expression in the Neonatal Rat. *Dev Neurosci, 25*(1), 26-33.

Kaplan, G. A., Cohn, B. A., Cohen, R. D., & Guralnik, J. (1988). The decline in ischemic heart disease mortality: prospective evidence from the Alameda County Study. *Am J Epidemiol, 127*(6), 1131-1142.

Kenny, D. A., Kashy, D. A., & Cook, W. L. (2006). *Dyadic data analysis*. New York, NY: Guilford.

Kiecolt-Glaser, J. K., Loving, T. J., Stowell, J. R., Malarkey, W. B., Lemeshow, S., Dickinson, S. L., et al. (2005). Hostile marital interactions, proinflammatory cytokine production, and wound healing. *Arch Gen Psychiatry, 62*(12), 1377-1384.

Kiecolt-Glaser, J. K., Malarkey, W. B., Chee, M. A., & Newton, T. L. (1993). Negative behaviour during marital conflict is associated with immunological down-regulation. *Psychosomatic Medicine, 55*(5), 395-409.

Kiecolt-Glaser, J. K., & Newton, T. L. (2001). Marriage and health: his and hers. *Psychol Bull, 127*(4), 472-503.

Kiistala, U. (1968). Suction blister device for separation of viable epidermis from dermis. *J Invest Dermatol, 50*(2), 129-137.

Kirsch, P., Esslinger, C., Chen, Q., Mier, D., Lis, S., Siddhanti, S., et al. (2005). Oxytocin modulates neural circuitry for social cognition and fear in humans. *J Neurosci, 25*(49), 11489-11493.

Kirschbaum, C., & Hellhammer, D. H. (1994). Salivary cortisol in psychoneuroendocrine research: recent developments and applications. *Psychoneuroendocrinology, 19*(4), 313-333.

Koivukangas, V. (2004). *Wound healing in a suction blister model.* University of Oulu, Oulu, Finland.

Kosfeld, M., Heinrichs, M., Zak, P. J., Fischbacher, U., & Fehr, E. (2005). Oxytocin increases trust in humans. *Nature, 435*(7042), 673-676.

Krohne, H. W., Egloff, B., Kohlmann, C.-W., & Tausch, A. (1996). Investigation with a German form of the Positive and Negative Affect Schedule (PANAS). *Diagnostica, 42,* 139-156.

Kuhns, D. B., DeCarlo, E., Hawk, D. M., & Gallin, J. I. (1992). Dynamics of the cellular and humoral components of the inflammatory response elicited in skin blisters in humans. *J Clin Invest, 89*(6), 1734-1740.

Landgraf, R., & Neumann, I. D. (2004). Vasopressin and oxytocin release within the brain: a dynamic concept of multiple and variable modes of neuropeptide communication. *Front Neuroendocrinol, 25*(3-4), 150-176.

Lauchli, S. (2007a). [Alternative methods for wound treatment]. *MMW Fortschr Med, 149*(46), 41-42. Lauchli, S. (2007b). Alternative and physical methods in wound treatment. *Hautnah, 5,* 266-271.

Malarkey, W. B., Kiecolt-Glaser, J. K., Pearl, D., & Glaser, R. (1994). Hostile behaviour during marital conflict alters pituitary and adrenal hormones. *Psychosom Med, 56*(1), 41-51.

McEwen, B. S. (1998). Protective and damaging effects of stress mediators. *N Engl J Med, 338*(3), 171-179.

Neumann, I. D. (2002). Involvement of the brain oxytocin system in stress coping: interactions with the hypothalamo-pituitary-adrenal axis. *Progress in Brain Research, 139,* 147-162.

Padgett, D. A., Marucha, P. T., & Sheridan, J. F. (1998). Restraint stress slows cutaneous wound healing in mice. *Brain Behav Immun, 12*(1), 64-73.

Parker, K. J., Buckmaster, C. L., Schatzberg, A. F., & Lyons, D. M. (2005). Intranasal oxytocin administration attenuates the ACTH stress response in monkeys. *Psychoneuroendocrinology, 30*(9), 924-929.

Raudenbush, S. W., & Bryk, A. S. (2002). *Hierarchical Linear Models: Applications and Data Analysis Methods*. Newbury Park , CA: Sage Publications.

Rief W. & Hiller W. (2008). *SOMS - Screening for Somatoform Disorders - Manual. 2nd, completely revised and newly standardised edition.* Bern: Huber-Verlag.

Robles, T. F., & Kiecolt-Glaser, J. K. (2003). The physiology of marriage: pathways to health. *Physiol Behav, 79*, 409-416.

Rohleder, N., Nater, U. M., Wolf, J. M., Ehlert, U., & Kirschbaum, C. (2004). Psychosocial stress-induced activation of salivary alpha-amylase: an indicator of sympathetic activity? *Ann N Y Acad Sci, 1032*, 258-263.

Rosen, R., Brown, C., Heiman, J., Leiblum, S., Meston, C., Shabsigh, R., et al. (2000). The Female Sexual Function Index (FSFI): a multidimensional self-report instrument for the assessment of female sexual function. *J Sex Marital Ther, 26*(2), 191-208.

Rosenblum, L. A., Smith, E. L., Altemus, M., Scharf, B. A., Owens, M. J., Nemeroff, C. B., et al. (2002). Differing concentrations of corticotropin-releasing factor and oxytocin in the cerebrospinal fluid of bonnet and pigtail macaques. *Psychoneuroendocrinology, 27*(6), 651-660.

Schaer, M., Ditzen, B., Heinrichs, M., & Bodenmann, J. G. (2007). Emotional and cardiovascular responses of couples during a couple therapy intervention. *Journal of Clinical Psychology and Psychotherapy, 36*(4), 251-260.

Schleimer, R. P., Claman, H. N., & Oronsky, A. L. (1989). *Anti-Inflammatory Steroid Action: Basic and Clinical Aspects*. San Diego, CA: Academic Press.

Schoenbach, V. J., Kaplan, B. H., Fredman, L., & Kleinbaum, D. G. (1986). Social ties and mortality in Evans County, Georgia. *Am J Epidemiol, 123*(4), 577-591.

Schulz, P., Schlotz, W., & Becker, P. (2004). *Das Trierer Inventar zur Erfassung von chronischem Stress - Version 2 (TICS 2) [Trier Inventory for the Assessment of Chronic Stress]*. Gottingen: Hogrefe.

Schumacher, J., Leppert, K., Gunzelmann, T., Strauss, B. & Brähler, E. (2004). The Resilience Scale, a questionnaire to assess psychological resilience as a personal characteristic [On-Line]. Available: http://www.mentalhealthpromotion.net/resources/resilienzskala2.pdf, as of July 2009.

Schwarzer, R & Schulze, U. (2000). Social support in coping with illness. The Berlin Social Support Scales (BSSS) [On-Line]. Available: http://userpage.fu-berlin.de/~health/materials/bsss.pdf, as of July 2009.

Steptoe, A., O'Donnell, K., Badrick, E., Kumari, M., & Marmot, M. (2008). Neuroendocrine and inflammatory factors associated with positive affect in healthy men and women: the Whitehall II study. *Am J Epidemiol, 167*(1), 96-102.

Steyer, R., Schwenkmezger, P., Notz, P., & Eid, M. (1997). *Der Mehrdimensionale Befindlichkeitsfragebogen (MDBF) [Multidimensional mood questionnaire]*. Göttingen: Hogrefe.

Taylor, S. E., Klein, L. C., Lewis, B. P., Gruenewald, T. L., Gurung, R. A., & Updegraff, J. A. (2000). Biobehavioural responses to stress in females: tend-and-befriend, not fight-or-flight. *Psychol Rev, 107*(3), 411-429.

Taylor, J. F., Rosen, R. C., & Leiblum, S. R. (1994). Self-report assessment of female sexual function: psychometric evaluation of the Brief Index of Sexual Functioning for Women. *Arch Sex Behav, 23*(6), 627-643

Test Centre (2008). [On-line]. Available: http://www.testzentrale.ch/de/neuheiten.php? choosenlang=en, as of July 2009.

Uvnas-Moberg, K. (1997). Oxytocin linked antistress effects--the relaxation and growth response. *Acta Physiol Scand Suppl, 640*, 38-42.

Uvnas-Moberg, K. (2003). *The Oxytocin Factor*: Da Capo Press. Vining, R. F., McGinley, R. A., Maksvytis, J. J., & Ho, K. Y. (1983). Salivary cortisol: a better measure of adrenal cortical function than serum cortisol. *Ann Clin Biochem, 20 (Pt 6)*, 329-335.

Wilkinson, R., & Marmot, M. (2003). *Social determinants of health: the solid facts* (2 ed.): World Health Organisation.

Williams, J. R., Carter, C. S., & Insel, T. (1992). Partner preference development in female prairie voles is facilitated by mating or the central infusion of oxytocin. *Ann N Y Acad Sci, 652*, 487-489.

Williams, R. B., Barefoot, J. C., Califf, R. M., Haney, T. L., Saunders, W. B., Pryor, D. B., et al. (1992). Prognostic importance of social and economic resources among medically treated patients with angiographically documented coronary artery disease. *Jama, 267*(4), 520-524.

Windle, R. J., Shanks, N., Lightman, S. L., & Ingram, C. D. (1997). Central oxytocin administration reduces stress-induced corticosterone release and anxiety behaviour in rats. *Endocrinology, 138*(7), 2829-2834.

Young, L. J. (2002). The Neurobiology of Social Recognition, Approach, and Avoidance. *Biol Psychiatry, 51*, 18-26. Young, L. J., & Wang, Z. (2004). The neurobiology of pair bonding. *Nat Neurosci, 7*(10), 1048-1054.
