# Supplementary material for: Intranasal Oxytocin and Physical Intimacy for Dermatological Wound Healing and Neuroendocrine Stress: A Randomized Clinical Trial
Source: JAMA Psychiatry. 2025 Nov 12;83(2):118–27. doi: 10.1001/jamapsychiatry.2025.3705 (PMC12613093; doi:10.1001/jamapsychiatry.2025.3705)
Supplement: Supplement 2. — eTable 1. Sociodemographic characteristics of the sample eTable 2. Oxytocin vs. placebo group differences in relationship and personality characteristics, and outcome variables eTable 3. PAT vs. nPAT group differences in relationship and personality characteristics, and outcome variables eFigure. Exemplary illustration of wound photographs on day 1, day 2, and day 7 [file jamapsychiatry-e253705-s002.pdf]

Supplemental Online Content

Schneider E, Hernández C, Brock R, et al. Intranasal oxytocin and physical intimacy for dermatological wound healing and neuroendocrine stress: a randomized clinical trial. *JAMA Psychiatry*. Published online November 12, 2025. doi:10.1001/jamapsychiatry.2025.3705

eTable 1. Sociodemographic characteristics of the sample

eTable 2. Oxytocin vs. placebo group differences in relationship and personality characteristics, and outcome variables

eTable 3. PAT vs. nPAT group differences in relationship and personality characteristics, and outcome variables

eFigure. Exemplary illustration of wound photographs on day 1, day 2, and day 7

This supplemental material has been provided by the authors to give readers additional information about their work.

© 2025 Schneider E et al. *JAMA Psychiatry*.

eTable 1: Sociodemographic characteristics of the sample

	Oxytocin Group (N = 80)								Placebo Group (N = 80)							
	PAT (N = 40)				Neutral (N = 40)				Positive (N = 38)				Neutral (N = 42)			
	M	*SD*	*Min*	*Max*	*M*	*SD*	*Min*	*Max*	*M*	*SD*	*Min*	*Max*	*M*	*SD*	*Min*	*Max*
Age	27.20	4.88	22	41	27.20	4.73	21	40	26.94	3.72	22	40	28.43	6.37	21	45

	Oxytocin PAT *n*	Oxytocin PAT *%*	Oxytocin Neutral *n*	Oxytocin Neutral *%*	Placebo Positive *n*	Placebo Positive *%*	Placebo Neutral *n*	Placebo Neutral *%*
Sex								
Male	20	*50.0*	20	*50.0*	19	*50.0*	21	*50.0*
Female	20	*50.0*	20	*50.0*	19	*50.0*	21	*50.0*
Hormonal Contraception[a]								
yes	10	*50.0*	10	*50.0*	9	*47.4*	11	*52.4*
no	10	*50.0*	10	*50.0*	10	*52.6*	10	*47.6*
Nationality								
Swiss	23	*57.5*	24	*60.0*	33	*86.8*	29	*69.05*
German	9	*22.5*	9	*22.5*	3	*7.9*	8	*19.05*
Austrian	2	*5.0*	-	-	-	-	1	*2.4*
Other	6	*15.0*	7	*17.5*	2	*5.3*	3	*7.1*
Missing	-	-	-	-	-	-	1	*2.4*
Education[b]								
Lower Secondary Education	-	-	-	-	-	-	2	*4.8*
Vocational training	-	-	2	*5.0*	2	*5.3*	5	*11.9*
Upper secondary academic education	16	*40.0*	*12*	*30.0*	8	*21.1*	15	*35.7*
Tertiary education degrees	23	*57.5*	*25*	*62.5*	26	*86.4*	18	*42.9*
Other	1	*2.5*	*1*	*2.5*	1	*2.6*	1	*2.4*
Missing					1	*2.6*	1	*2.4*
Income								
No income	-	-	*1*	*2.5*	-	-	1	*2.4*
Student	17	*42.5*	*16*	*40.0*	18	*47.4*	14	*33.3*
≤ 50.000 Swiss francs/year	11	*27.5*	*4*	*10.0*	8	*21.1*	17	*40.5*
≤ 100.000 Swiss francs/year	8	*20.0*	*13*	*32.5*	9	*23.7*	9	*21.4*
>100.000 Swiss francs/year	4	*10.0*	*6*	*15.0*	2	*5.3*	-	-
Missing	-	-	-	-	1	*2.6*	1	*2.4*
Employment								
Yes	36	*90.0*	*29*	*72.5*	31	*81.6*	31	*73.8*
No	4	*10.0*	*11*	*27.5*	6	*15.8*	10	*23.8*
Missing	-	-	-	-	1	*2.6*	1	*2.4*

Note. [a] counting only female participants

[b] In this table, educational attainment was classified into four categories based on the Swiss education system: (1) Lower secondary education, including lower secondary school diplomas (e.g., Realschulabschluss, Sekundarabschluss); (2) Vocational training qualifications, including apprenticeship diplomas (Lehrabschluss); (3) Upper secondary academic education, represented by the Swiss matriculation certificate or vocational baccalaureate (Matura/Kantonschulabschluss, Berufsmatura); and (4) Tertiary education, including university degrees at the Bachelor's, Master's, and Doctoral levels.

© 2025 Schneider E et al. *JAMA Psychiatry*.

eTable 2. Oxytocin vs. Placebo group differences in relationship and personality characteristics, and outcome variables

Relationship and Personality Characteristics	Oxytocin Group (n = 80) Mean (SD)	Placebo Group (n = 80) Mean (SD)	t	p	Cohen's d
Relationship duration (in years)*	4.10 (2.75)	3.72 (2.52)	-.58	.56	-.13
Cohabitation duration (in years)*	2.06 (1.96)	1.99 (1.67)	-.17	.86	-.04
Partnership quality (PFB)	71.19 (7.43)	72.81 (9.54)	1.20	.23	.19
NEO-FFI					
Extraversion	2.66 (.56)	2.50 (.58)	-1.70	.09	-.27
Conscientiousness	3.08 (.56)	3.12 (.46)	.53	.60	.08
Neuroticism	1.24 (.70)	1.23 (.70)	-.06	.95	-.01
Openness to Experience	2.64 (.76)	2.80 (.70)	1.34	.18	.21
Agreeableness	2.98 (.55)	2.97 (.53)	-.08	.93	-.01
**Outcome Variables**					
Wound severity T1	4.36 (.87)	4.37 (.97)	.10	.92	.02
Wound severity T2	3.91 (.73)	4.00 (.44)	.99	.33	.16
Wound severity T3	.99 (1.21)	.96 (1.11)	-.17	.86	-.03
Affectionate touch	7.78 (4.72)	9.26 (5.49)	1.84	.07	.29
Sexual activity	1.45 (1.27)	1.36 (1.40)	-.41	.68	-.07
Subjective stress	1.28 (.44)	1.23 (.49)	-.65	.52	-.10
Cortisol AUCg	6291.48 (2874.80)	5882.50 (2405.69)	-.97	.33	-.15

eTable 3. PAT vs. nPAT group differences in relationship and personality characteristics, and outcome variables

Relationship and Personality Characteristics	PAT Group (n = 78) Mean (SD)	nPAT Group (n = 82) Mean (SD)	t	p	Cohen's d
Relationship duration (in years)*	3.99 (2.54)	3.80 (2.74)	-.33	.74	-.07
Cohabitation duration (in years)*	2.22 (1.80)	1.84 (1.82)	-.94	.35	-.21
Partnership quality (PFB)	72.24 (8.72)	71.77 (8.46)	-.35	.73	-.06
NEO-FFI					
Extraversion	2.64 (.57)	2.52 (.58)	-1.30	.20	-.21
Conscientiousness	3.05 (.54)	3.14 (.48)	1.22	.23	.19
Neuroticism	1.20 (.68)	1.26 (.71)	.53	.60	.09
Openness to Experience	2.65 (.70)	2.79 (.75)	1.20	.23	.19
Agreeableness	2.94 (.52)	3.00 (.56)	.68	.50	.11
**Outcome Variables**					
Wound severity T1	4.51 (.87)	4.22 (.96)	-1.91	.06	-.31
Wound severity T2	3.99 (.68)	3.91 (.51)	-.84	.40	-.13
Wound severity T3	1.02 (1.17)	.93 (1.15)	-.51	.61	-.08
Affectionate touch	7.38 (5.22)	9.59 (4.89)	2.77	.006	.44
Sexual activity	1.33 (1.24)	1.48 (1.42)	.67	.50	.11
Subjective stress	1.19 (.49)	1.32 (.42)	1.90	.06	.30
Cortisol AUCg	5797.58 (1813.14)	6357.05 (3236.44)	1.33	.19	.21

Note. Wound severity reflects ratings based on the revised Photographic Wound Assessment Tool (revPWAT). T1 = 1 hour, T2 = 24 hours, T3 = 7 days after wound application. Affectionate touch and sexual activity scores represent daily sums across the EMA period. Subjective stress ratings and cortisol AUCg values reflect individual mean scores across EMA. * Group differences on relationship duration and cohabitation duration were analyzed based on one value per couple; all other characteristics were analyzed, including individual values.

© 2025 Schneider E et al. *JAMA Psychiatry*.

eFigure. Exemplary illustration of wound photographs on day 1 (1), day 2 (2), and day 7 (3).

ID 43 Male

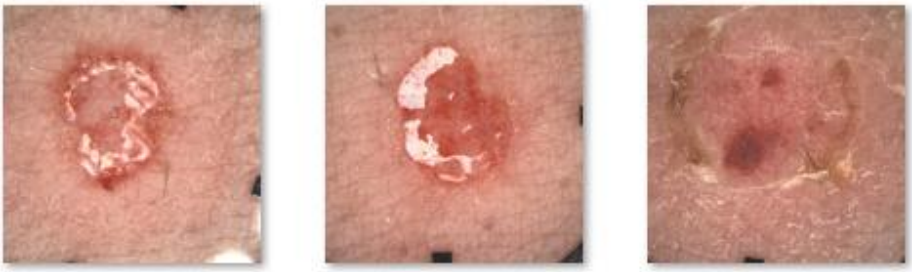

1 2 3

ID 49 Female

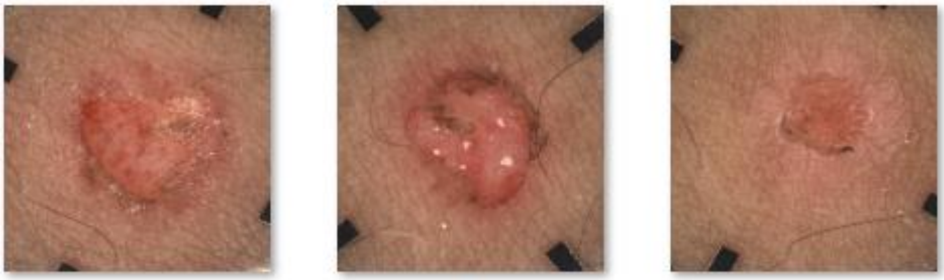

1 2 3

ID 65 Female

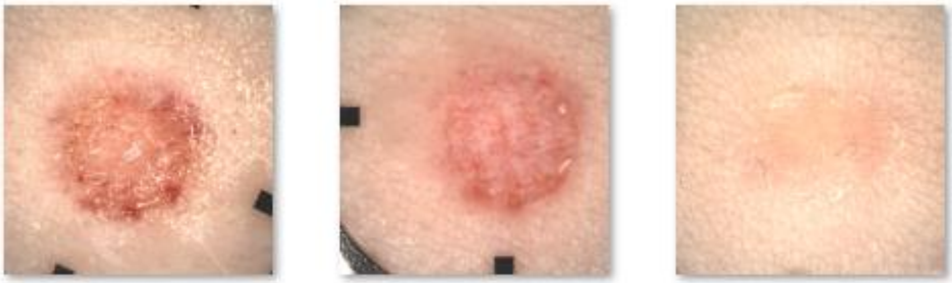

1 2 3

ID 58 Male

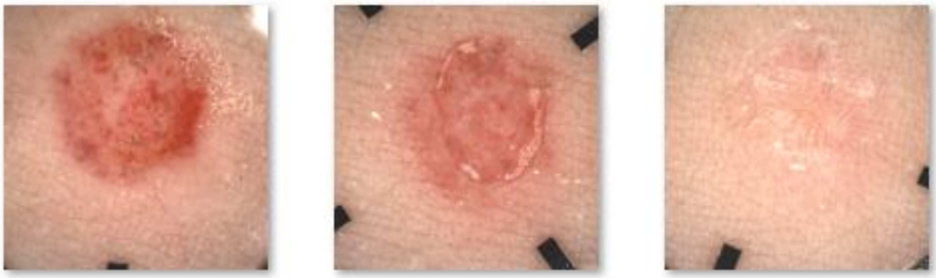

1 2 3

© 2025 Schneider E et al. *JAMA Psychiatry*.
