# Supplementary material for: Intranasal Oxytocin and Physical Intimacy for Dermatological Wound Healing and Neuroendocrine Stress: A Randomized Clinical Trial
Source: JAMA Psychiatry. 2025 Nov 12;83(2):118–27. doi: 10.1001/jamapsychiatry.2025.3705 (PMC12613093; doi:10.1001/jamapsychiatry.2025.3705)
Supplement: Supplement 3. — Data sharing statement [file jamapsychiatry-e253705-s003.pdf]

Data Sharing Statement

Schneider. Intranasal Oxytocin and Physical Intimacy for Dermatological Wound Healing and Neuroendocrine Stress. *JAMA Psychiatry*. Published November 12, 2025. doi:10.1001/jamapsychiatry.2025.3705

Data
Additional Information: ClinicalTrials.gov identifier: NCT01594775
Data available: No

Additional Information
Explanation for why data not available: Data will be provided upon request.
